# Food Additives as Microbiome-Active Dietary Exposures: Mechanisms, Host Susceptibility, and an Operational Case for Microbiome-Inclusive Risk Assessment

**DOI:** 10.3390/nu18213461

**Published:** 2026-10-22

**Authors:** Ioana Cristina Crivei, Roxana Nicoleta Ratu, Ionuț-Dumitru Veleșcu, Andreea Bianca Balint, Florin Daniel Lipșa, Florina Stoica, Luciana Alexandra Crivei

**Affiliations:** 1 Department of Food Technologies, Faculty of Agriculture, “Ion Ionescu de la Brad” Iasi University of Life Sciences, 3 Mihail Sadoveanu Alley, 700489 Iasi, Romania; ioana.crivei@iuls.ro (I.C.C.); roxana.ratu@iuls.ro (R.N.R.); bianca.balint@iuls.ro (A.B.B.); florin.lipsa@iuls.ro (F.D.L.); 2 Research Institute for Agriculture and Environment, “Ion Ionescu de la Brad” Iasi University of Life Sciences, 14 Sadoveanu Alley, 700490 Iasi, Romania; florina.stoica@iuls.ro; 3 Department of Public Health, Faculty of Veterinary Medicine, “Ion Ionescu de la Brad” Iasi University of Life Sciences, 3 Mihail Sadoveanu Alley, 700490 Iasi, Romania; luciana.crivei@iuls.ro

**Keywords:** food additives, gut microbiota, microbiome, emulsifiers, non-nutritive sweeteners, intestinal barrier, immune modulation, risk assessment, ultra-processed foods, host susceptibility

## Abstract

Food additives are defining components of ultra-processed foods, yet microbiome-related effects remain largely outside routine safety assessment. This review evaluates evidence for six additive classes across four pathways—microbial community changes, altered microbial metabolites, mucus-epithelial barrier disruption, and immune modulation—and examines how these findings could inform microbiome-inclusive risk assessment. Mechanistic, translational, and epidemiological evidence was organized using a five-tier hierarchy: in vitro, ex vivo, animal, observational, and controlled human studies. Chronic-disease associations were graded across six domains by two raters using explicit criteria, with sensitivity analysis. Evidence was then translated into a tiered, decision-gated assessment protocol, illustrated with carboxymethylcellulose and back-tested against carrageenan, acesulfame K, and titanium dioxide, which return three different protocol outcomes. Non-nutritive sweeteners and emulsifiers have the strongest direct human and ex vivo evidence, including microbiome-dependent interindividual responses, although null and discordant trials remain. Gastrointestinal and metabolic outcomes received the highest support, cardiovascular associations were intermediate, and autoimmune, neurological, and psychiatric links remained preliminary. Metabolic and cardiovascular epidemiology relied largely on NutriNet-Santé, with modest hazard ratios (generally 1.03–1.15 per exposure increment) and indirect exposure estimation. Recent additive-mixture analyses from the same cohort were also considered. Food additives can act as context-dependent, microbiome-active exposures. Current evidence supports considering microbiome composition, barrier-integrity, and immune endpoints in additive safety evaluation, while causal links with most chronic-disease outcomes remain unproven.

## 1. Introduction

The composition of the global food supply has shifted markedly over recent decades as minimally processed foods have given way to industrially formulated products. Ultra-processed foods (UPFs), as defined by the NOVA classification, are industrial formulations built around additives—emulsifiers, non-nutritive sweeteners (NNS), preservatives, colorants, and texture modifiers—used to extend shelf life and to tune palatability, stability, and sensory appeal [1,2,3]. National intake surveys and global sales data indicate that UPF-based dietary patterns are now dominant in both high-income and nutritional-transition economies [2].

The epidemiological signal linking UPF consumption to poor health is broad. In an umbrella review of 45 meta-analyses covering more than 9.9 million participants, Lane et al. reported associations between higher UPF intake and elevated risks of cardiovascular mortality, type 2 diabetes, common mental disorders, all-cause mortality, and obesity [1]. These associations derive mostly from observational designs and remain open to residual confounding by dietary quality, socioeconomic status, and physical activity. Their consistency across independent cohorts and outcome domains is nonetheless compatible with a population-level dietary risk that macronutrient content alone does not explain. Two further lines of evidence support that reading. In a controlled dietary trial, participants on an unprocessed diet spontaneously reduced energy intake while eating larger meals of lower energy density [4], and a synthesis of more than 100 prospective studies, randomized controlled trials (RCTs), and mechanistic investigations concluded that UPF-based patterns promote excess energy intake through processing-related features that macronutrient profiling does not capture [2]. Together, these findings motivate the hypothesis that non-macronutrient features of UPFs, among them food additives but also physical processing and changes to the food matrix, have biological effects that current nutritional frameworks are not designed to detect.

Gut microbiome science offers a mechanism for how additives might reach host physiology. The gut microbiota, whose collective metabolic capacity far exceeds that of the human genome, sits at the interface between diet and systemic biology [5]. Multi-omics work has linked specific microbial pathways—short-chain fatty acid (SCFA) production, bile acid transformation, tryptophan catabolism, and generation of immune-modulating metabolites—to cardiometabolic, inflammatory, and neurological outcomes [5]. Changes in microbial diversity, composition, and function (collectively, dysbiosis) accompany low-grade inflammation, insulin resistance, gastrointestinal dysfunction, and immune dysregulation, though the direction and causal structure of these relationships are still being worked out [6].

Within this framework, food additives have emerged as a biologically relevant but under-characterized group of microbiome-active exposures. The emulsifiers carboxymethylcellulose (CMC, E466) and polysorbate-80 (P80, E433) are the best-studied examples. Ex vivo screening showed that the capacity to reshape community structure and metabolite output is shared across structurally different emulsifiers, with compound-specific profiles [7], and in mice several emulsifiers produced dysbiosis alongside intestinal inflammation and features of metabolic syndrome [8]. In a randomized controlled-feeding trial, healthy adults consuming CMC at dietary-relevant doses developed altered microbiota composition, shifted metabolomic profiles and, in a subset, microbiota encroachment into the inner mucus layer [9]; responses varied with baseline microbiome configuration, and an in vitro microbiota reactor subsequently showed that baseline metagenomic features predict individual sensitivity to emulsifier exposure [10]. Suez et al. found that saccharin, sucralose, aspartame and stevia glycosides produced person-specific, microbiome-dependent glycemic responses partly predictable from baseline gut and oral microbiota [11]. Host susceptibility is therefore tractable for these two classes and offers a biological reading of the inconsistent NNS literature. Not every human trial finds an effect: a within-ADI crossover RCT of aspartame and sucralose reported no significant compositional change [12], and such null results are addressed directly rather than explained away. Beyond these classes, maltodextrin impaired mucus-barrier function and accelerated colitis in susceptible mice through mechanisms independent of direct antimicrobial activity [13]; broad syntheses identify dysbiosis, barrier impairment and immune activation as recurring themes across structurally heterogeneous compounds [6,14]; and comparative fermentation shows that emulsifiers, sweeteners and preservatives act in compound-specific rather than class-uniform ways, which argues for evaluating individual compounds [15].

Despite this progress, the evidence base remains fragmented. Preclinical exposure concentrations often exceed estimated human intake; controlled human data are few, short, and small; and epidemiological studies struggle to isolate the contribution of any single additive within complex UPF patterns [6]. Additives are also rarely eaten alone: a single UPF product routinely carries several co-occurring additives whose combined effects were, until recently, almost unstudied on the population scale. Current regulatory frameworks from the European Food Safety Authority (EFSA) and the Food and Drug Administration (FDA) rely on conventional toxicological endpoints and do not routinely assess microbiome composition, epithelial barrier integrity, or immune modulation, so acceptable daily intakes (ADIs) may miss microbiome-mediated effects that operate below organ-toxicity thresholds, particularly in people with distinctive baseline configurations [6].

### Positioning Relative to Existing Reviews and Regulatory Activity, and the Contribution of This Work

Several recent reviews cover parts of this landscape [16,17,18,19,20,21]. The most comprehensive, Whelan et al. [6], catalogs additive effects on gut health largely by additive class. In parallel, the regulatory science has moved: the European Food Safety Authority has published a roadmap for integrating gastro-intestinal tract microbiomes into risk assessments under its remit [22] and has issued guidance on the characterization and risk assessment of microorganisms in the food chain [23], building on earlier “toxicomicrobiomics” work [24]. The idea that microbiome endpoints belong in food-safety evaluation is therefore no longer novel in itself; the gap is operational.

This review is deliberately positioned against that background, and its contribution is integrative and normative rather than a claim to new data. It differs from prior work in four respects, set out explicitly and compared item by item in Table 1. First, the synthesis is organized by biological pathway rather than by additive class, so that convergent mechanisms operating across heterogeneous compounds become visible. Second, baseline microbiome configuration is treated as an explanatory axis for inter-individual variability, but only where direct evidence exists (NNS and emulsifiers); for the other classes it is presented as a hypothesis to be tested. Third, and most substantively, the EFSA roadmap’s general call is turned into an additive-specific, decision-gated protocol: a tiered sequence of screens with explicit pass/fail criteria, a defined minimum endpoint set, and a single additive (CMC) worked through it as a proof of concept. Fourth, an explicit, anchored grading of six chronic-disease domains is applied; association strength, mechanistic plausibility, and causal confidence are rated separately; and a sensitivity analysis is reported so that the grades can be interrogated rather than taken on trust.

No single one of these elements is claimed to be unprecedented. The intended contribution is the combination applied to a concrete regulatory question, the operational detail of the proposed protocol, and the discipline of separating what the evidence supports from what it does not.

## 2. Literature Search and Evidence Synthesis Approach

This is a narrative, integrative review. A systematic review was not performed, and no claim of exhaustive retrieval is made; accordingly, no PRISMA flow diagram is presented, nor is PRISMA used as a completeness warrant. Reporting follows the Scale for the Assessment of Narrative Review Articles (SANRA) [26], appropriate for an integrative synthesis of a heterogeneous, fast-moving field [27]. The aim was to assemble the most informative mechanistic, translational, observational, and regulatory evidence on additive–microbiota–host interactions and to appraise it transparently by evidence tier. Although narrative in design, the review adopts three reporting practices from systematic-review methodology to increase rigor and reproducibility without claiming systematic-review status: the search strategies are documented in full per the PRISMA-S extension [28] (Text S1); the pivotal observational studies are appraised for risk of bias with ROBINS-E [29], the instrument appropriate to observational studies of exposures (Table S2 and Text S2); and the certainty of evidence for the principal exposure–outcome relationships is rated per outcome with GRADE [30] (Table 2). The qualitative synthesis follows a direction-of-effect, SWiM-style approach [31] rather than meta-analysis, given the heterogeneity of designs and endpoints.

### 2.1. Databases, Scope, and Search Terms

Four databases were searched: PubMed/MEDLINE, Embase (Ovid), Scopus, and Web of Science Core Collection. The primary window was January 2020 to June 2026, to emphasize recent evidence; earlier publications were retained only where they were foundational and had no 2020-or-later equivalent. English-language articles were searched with no restriction on country or journal; preprints were neither searched nor cited. Gray literature, conference proceedings, theses, and trial registries were likewise not searched systematically, although EFSA, JECFA, and FDA documents were hand-searched as described in Text S1. Restriction to English-language, peer-reviewed, published records is a source of publication and language bias. Citations were also chained from high-quality reviews to capture additional primary studies [32]. The full Boolean strategies for each database, with platform, coverage, date searched, and filters, are provided in Text S1, reported in line with the PRISMA-S search-reporting extension [28].

Search terms were drawn from four conceptual domains, combined within each domain by OR, with MeSH adaptation for PubMed. Because combining more than three concept blocks suppresses sensitivity, the four domains were not all intersected in a single query: the primary strategy combined Domain I (exposure) AND Domain II (microbiota), and focused variants added Domain III or Domain IV as a third block. Text S1 reports the executed strings for each platform. The domains were: (I) additive exposure (“food additives”, “dietary emulsifiers”, “non-nutritive/artificial sweeteners”, and named compounds including carboxymethylcellulose, polysorbate-80, carrageenan, maltodextrin, titanium dioxide, sodium benzoate, sucralose, and aspartame, plus “ultra-processed food”); (II) gut microbiota (“gut/intestinal microbiome”, “microbial diversity”, “dysbiosis”, “short-chain fatty acids”, “bile acid/tryptophan metabolism”); (III) host mechanisms (“intestinal barrier”, “tight junction”, “mucus layer”, “innate immunity”, “inflammasome”, “NF-κB”, “gut–brain axis”); and (IV) health outcomes (“inflammatory bowel disease”, “metabolic syndrome”, “type 2 diabetes”, “cardiovascular disease”, “colorectal cancer”, “obesity”, “autoimmune/neurological disorders”). Table S1 lists the full-term set and the inclusion and exclusion criteria. Compound- and class-specific searches were run for each of the six classes, with additional targeted searches on host susceptibility, mixture effects, regulatory guidance, and evidence tiering.

### 2.2. Selection and Data Extraction

Records were deduplicated and screened by title and abstract against predefined criteria, then read in full for microbiome-endpoint reporting, dose-relevance to human exposure, design clarity, and a direct link to additive exposure. Screening and extraction were performed by the authors. Screening was not duplicated independently: one author screened titles and abstracts and a second author verified the included set and adjudicated uncertain records, so no inter-screener agreement statistic can be reported. Single screening is known to miss eligible records relative to duplicate independent screening, and no formal search-peer-review (PRESS) was obtained. The study types included are inventoried in Text S4. These are limitations of retrieval completeness rather than of the appraisal that follows, but they constrain how strongly any conclusion about the completeness of the evidence base can be put.

### 2.3. A Five-Tier Evidence Hierarchy with Explicit Translation Limits

Evidence is appraised against a five-tier hierarchy that states what each tier can and cannot establish, adapted from translational research and regulatory toxicology [24,27] to the specifics of microbiome science, where in-vitro-to-human translation is complicated by inter-individual variability, species-specific ecology, and chronic mixed exposure [33,34].

Tier 1—In vitro (cell lines, organoids, batch fermentation) establishes direct, mechanistically specific effects under controlled, dose-defined conditions and supports high-throughput screening and pathway identification. It cannot establish physiological relevance under gut transit, mixture behavior from single-additive data, or that simplified communities reflect the human microbiota; alone it does not support clinical or regulatory conclusions [33].

Tier 2—Ex vivo (human fecal incubations, mucosal biopsies, SHIME—the Simulator of the Human Intestinal Microbial Ecosystem) incorporates genuine human microbial diversity and can establish community and metabolite effects within realistic ecosystems, but cannot capture host-microbiota signaling, mucosal immunity, transit dynamics, or digestive transformation [33,34].

Tier 3—Animal models (conventional, germ-free, antibiotic-depleted, humanized) establish in vivo causal links between exposure and microbiome change, barrier dysfunction, immune activation, and metabolic outcomes; germ-free and antibiotic-attenuation designs give the strongest experimental evidence for microbiome-mediated causation. Translation requires attention to species-specific microbiota, dose-to-human scaling, and the genetic homogeneity of inbred strains; humanized models address species-specificity only partly [35].

Tier 4—Human observational (cohort, cross-sectional, case–control) establishes associations under realistic exposure but cannot establish causation, isolate specific additives from correlated dietary variables, or fully control residual confounding; large cohorts such as NutriNet-Santé provide the strongest current Tier 4 evidence, subject to the exposure-measurement and selection limits addressed later.

Tier 5—Controlled human intervention (randomized feeding or crossover trials) gives the strongest human causal evidence but is limited in number, duration (usually ≤4 weeks), sample size, and compound breadth; individual response heterogeneity, partly attributable to baseline configuration, is a defining feature that larger trials will be needed to resolve at the subgroup level.

Throughout, each finding is assigned to its tier. Where a Tier 1/2 mechanism is supported by Tier 3 causal models and Tier 5 human evidence, the conclusion is treated as higher confidence. Where tiers disagree, dose-translation failure, species-specificity, or susceptibility heterogeneity are considered as candidate explanations; susceptibility heterogeneity is not, however, treated as a default explanation for every discordant result, because doing so would make the thesis unfalsifiable. At the level of the whole model, a matching test is available: the pathway account predicts that an additive without a compositional, metabolite, or barrier signal should not carry a consistent disease association, so a robust additive–disease association in a compound that is inert on the Tier A screen described below would count as evidence against it.

### 2.4. Operationalised Grading Across Six Chronic-Disease Domains

Evidence is graded across six domains (gastrointestinal, metabolic, cardiovascular, autoimmune, neurological, psychiatric) on three dimensions rated independently. To make the grading reproducible rather than impressionistic, each level is defined by explicit anchors.

Strength of association—Strong: consistent, replicated associations in ≥2 independent large prospective cohorts or a meta-analysis, with effect estimates in a consistent direction; Moderate: associations in a single prospective cohort or several cross-sectional studies with some inconsistency; Weak: sparse, cross-sectional, or inconsistent associations; Absent: no human association data.

Mechanistic plausibility—Strong: a coherent pathway supported by Tier 3 causal models with converging Tier 1/2 data; Moderate: Tier 1–3 support with gaps or model-dependence; Emerging: fragmentary or indirect mechanistic data; Speculative: hypothesized only.

Causal confidence—High: cross-tier convergence including Tier 5 human evidence on the relevant clinical endpoint; Moderate: Tier 5 evidence on surrogate endpoints or strong Tier 3 causal data; Low: predominantly animal or observational; Insufficient: not gradable.

The anchors were applied independently by two of the authors after full extraction; the raters were therefore not independent of the review’s hypothesis. Raw agreement before consensus was 15 of 18 cells (83%); the three discordant cells each differed by one adjacent level and were resolved by discussion. Given the small number of ordinal cells, a chance-corrected κ was not computed, as it would be unstable at this scale; raw percentage agreement correspondingly overstates reliability when ratings cluster on a few ordinal levels, as they do here. Both raters were also co-authors and so were not blinded to the review’s argument. The grading is therefore presented as structured expert judgment rather than as a validated instrument. An external rater blinded to the manuscript, or duplicate rating with weighted κ on a larger cell set, would be required before these grades could be treated as a reproducible instrument rather than as the authors’ structured reading of the evidence. The anchors were applied uniformly across domains; the gastrointestinal association is graded Moderate on that basis (Section 8.1). A sensitivity analysis is also reported in which moving any single cell by one level does not change which domains fall into the “most mature”, “intermediate”, and “preliminary” tiers. Where evidence was insufficient to grade a dimension, this is stated. The resulting matrix presented in Section 8, separates domains by current evidentiary maturity. Crucially, these labels describe the state of the evidence, not a recommendation for regulatory or clinical action; the distinction is developed below. This addresses a real weakness of prior syntheses, which tend to present associations across all outcomes with equal weight despite large differences in evidence quality [6,24].

To connect this domain-level grading to a standard certainty framework, the certainty of evidence for the principal exposure–outcome relationships was additionally rated per outcome using GRADE [30]. Following GRADE, observational bodies of evidence started at low certainty and were down- or up-graded on the standard factors (risk of bias, inconsistency, indirectness, imprecision, publication bias; large effect, dose–response); the resulting Summary-of-Findings judgements are given in Table 2, which lists the downgrading factors that applied to each relationship. Because observational evidence enters GRADE at Low certainty, the application of two or three serious concerns leaves most rows at Low rather than moving them further: the floor is reached quickly. Only the non-nutritive-sweetener row, where inconsistency across trials compounds risk of bias and indirectness, was taken below Low. No row was upgraded, since neither a large effect nor a documented dose–response on a clinical endpoint was available. Consistent with this, the pivotal observational cohorts were appraised for risk of bias with ROBINS-E [29] across its seven domains (Table S2 and Text S2). GRADE certainty is, by construction, conservative for observational evidence and is reported alongside—not in place of—the three-dimensional matrix, which conveys mechanistic and associational information that a single certainty label does not. Where the two frameworks appear to diverge (for example, a domain rated Moderate causal confidence in the matrix but Low in GRADE), the difference is definitional rather than contradictory: the matrix credits convergent mechanistic and surrogate-endpoint evidence, whereas GRADE certainty refers to the hard clinical endpoint. For any inference about causation of incident disease, the more conservative GRADE certainty takes precedence.

**Table 2:** GRADE Summary of Findings for the principal exposure–outcome relationships. Certainty was rated per outcome by standard GRADE [30], with observational evidence starting at “low”; ratings are deliberately conservative. Table 2 grades individual exposure–outcome pairs, whereas Table 5 maps evidence maturity across disease domains—the two are complementary, not redundant. RoB = risk of bias (ROBINS-E, Table S2).

Exposure → Outcome	Evidence Base	Main Downgrading Factors	GRADE Certainty	Plain-Language Statement
Emulsifiers → incident T2D	1 prospective cohort (NutriNet, *n* = 104,139; HR 1.03–1.15 per increment) + Tier 1–3 mechanistic	RoB (exposure measurement: ROBINS-E High); imprecision (modest effects); indirectness (single cohort)	Low	Dietary emulsifiers may increase the risk of type 2 diabetes; the certainty is low.
Emulsifiers → cardiovascular disease	1 cohort (NutriNet, *n* = 95,442; HR ≈ 1.05–1.11) + mechanistic	RoB (exposure measurement); indirectness (single cohort; no RCT)	Low	Dietary emulsifiers may increase cardiovascular risk; the certainty is low.
Non-nutritive sweeteners → incident T2D	1 cohort + a glycaemic-surrogate RCT [11] + a null microbiome RCT [12]	RoB; inconsistency (null/discordant trials); indirectness (surrogate endpoints)	Low to Very low	Non-nutritive sweeteners may increase the risk of type 2 diabetes; the evidence is of low-to-very-low certainty and is inconsistent across trials.
Additive mixtures → incident T2D	1 cohort (NutriNet, *n* = 108,643; HR 1.08–1.13 per 1 SD) [36]	RoB; single cohort; data-driven exposure definition	Low	Certain co-occurring additive mixtures may increase the risk of type 2 diabetes; the certainty is low.
Emulsifiers/carrageenan → IBD activity	Tier 3 causal models + case–control [37] + dietary-exclusion evidence [38]	Indirectness; sparse controlled human data	Low (moderate mechanistic plausibility)	Emulsifiers may contribute to inflammatory bowel disease activity; the certainty is low, with stronger mechanistic support than for other domains.
Additives → neurological/psychiatric outcomes	Predominantly animal models, often supra-physiological doses	RoB; serious indirectness (species, dose); very sparse human data	Very low	The effect of food additives on neurological and psychiatric outcomes is very uncertain.

## 3. Classification of Food Additives Relevant to the Gut Microbiota

Classification by technological function and E-number is necessary for labeling and safety dossiers but is poorly suited to mechanistic synthesis: additives that share a function may act through different pathways, while structurally unrelated additives from different classes may converge on the same host–microbiome interface. A dual scheme is therefore used—additives are grouped first by regulatory and technological category, then by their primary biological interaction mode—as summarized in Table 3, which also states the evidence tier behind each grouping [17,25]. Six classes with documented or credible microbiome activity are covered.

### 3.1. Non-Nutritive Sweeteners

NNS (saccharin, sucralose, aspartame, acesulfame K, stevia glycosides) are among the most widely used additives, spanning beverages, tabletop sweeteners, dairy, and reduced-calorie foods. Their primary mode is functional modulation of microbial metabolic output and compositional balance, through interactions with carbohydrate-transport systems, changes in fermentable-substrate availability, and compound-specific effects on key fermenting taxa [11,44]. Their defining feature is high inter-individual variability in host response, partly predictable from baseline composition, which makes NNS the class most directly relevant to the host-susceptibility axis. The most informative human evidence is the Suez et al. RCT, in which two weeks of supplementation at one to three servings per day produced person-specific, microbiome-dependent glycemic responses whose direction was partly predictable from baseline gut and oral microbiota [11]; this reframes some discordant prior findings as susceptibility heterogeneity rather than contradiction. That reframing has limits, and those limits are imposed here: a within-ADI crossover RCT of aspartame and sucralose in healthy adults found no significant change in microbiome composition [12], and sweetener–microbiome effects across the wider trial literature are mixed. NNS are therefore treated as a class where baseline-dependent responses are real but neither universal nor uniform. Comparative fecal batch fermentation shows that sucralose-, saccharin-, and aspartame-based products have compound-specific effects on abundance and fiber fermentation, so class-level generalization is not warranted [15]; additional in vitro work indicates concentration-dependent inhibition of commensals including *Lactobacillus* and *Bifidobacterium*, a secondary antimicrobial mode [44]. NNS are designated as a high-priority class: it has a credible, experimentally supported mechanism, reproducible evidence for baseline-attributable response heterogeneity, and an evidence base spanning human supplementation trials, controlled microbial systems, and observational epidemiology.

Aspartame also carries regulatory context that bears on any health framing. In 2023 the International Agency for Research on Cancer classified it as possibly carcinogenic to humans (Group 2B), while the Joint FAO/WHO Expert Committee on Food Additives maintained the existing ADI of 40 mg/kg body weight per day [45]. The Group 2B classification reflects limited evidence and is not a statement about the microbiome pathways reviewed here, but it should be acknowledged alongside them. That regulatory frameworks revisit their conclusions is not hypothetical: EFSA re-evaluated acesulfame K in 2025 and revised its acceptable daily intake [46], illustrating that additive evaluations are periodically reopened as evidence accumulates.

### 3.2. Emulsifiers

Emulsifiers (CMC E466; P80 E433; mono- and diglycerides E471; lecithin E322; sucrose fatty-acid esters; carrageenan E407) are amphiphilic molecules that stabilize emulsions, extend shelf life, and modify texture. Their primary mode is disruption of the mucus–epithelial interface: surfactant-like emulsifiers solubilize mucin, thin the mucus layer, promote bacterial translocation toward the epithelium, and alter luminal ecology through changed nutrient gradients [9,10,25]. This class has the most complete translational evidence chain—ex vivo human fecal incubation, controlled animal exposure, and controlled human feeding. Naimi et al. established the class-level nature of emulsifier effects by screening a structurally diverse panel in ex vivo human fecal cultures, showing that shifts in community structure and SCFA profiles are a shared property with compound-specific magnitudes [7]. Panyod et al. provided in vivo confirmation across lecithin, sucrose fatty-acid esters, CMC, and mono-/diglycerides, each inducing metabolic disorder and dysbiosis at moderate dietary intake [8]. Human translation came from a randomized controlled-feeding trial in which adults consuming CMC over eleven days showed reduced diversity, community shifts, fecal metabolomic changes consistent with reduced fermentation, including depletion of short-chain fatty acids and free amino acids, and, in a subset, microbiota encroachment into the normally sterile inner mucus layer [9]; the individual heterogeneity was partly explained by baseline configuration, which Rytter et al. extended with an in vitro reactor showing that baseline metagenomic features predict individual sensitivity [10]. Cox et al. reviewed emulsifier legislation and safety assessment and noted that current evaluation does not incorporate microbiome-relevant endpoints [25]. Emulsifiers are a high-priority class and the main exemplars of the mucus-epithelial pathway.

Carrageenan (E407) warrants a specific caveat, because much of the lay and some of the scientific literature conflates two distinct materials. Food-grade carrageenan is a high-molecular-weight polymer; poligeenan (formerly “degraded carrageenan”) is a low-molecular-weight acid-hydrolyzed product with a different toxicological profile, not permitted as a food additive [47]. EFSA’s 2018 re-evaluation retained a temporary ADI for carrageenan and flagged the low-molecular-weight fraction as the principal uncertainty, recommending that it be minimized [48]. Claims about carrageenan harm should therefore specify which material is meant and what molecular-weight distribution was tested.

### 3.3. Preservatives

Preservatives are a chemically diverse group designed to inhibit microbial growth: organic-acid salts (sodium benzoate E211, potassium sorbate E202, sodium propionate E281), nitrites and nitrates (E250, E251), and sulphiting agents (E220, E223). Their primary mode—direct antimicrobial pressure—follows from their technological purpose, which gives a high prior probability of microbiome effects; the open questions concern magnitude, compound specificity, dose-dependence, and how much in-food concentration survives gastric transit [39]. Li et al. exposed healthy mice to eleven preservatives spanning biogenic (nisin, natamycin) and synthetic (benzoic acid, sorbic acid, sodium nitrite, sulfur dioxide) agents at dietary-range doses and found compound-specific perturbations that did not track a biogenic-versus-synthetic split, challenging the assumption that naturally derived preservatives are microbiologically neutral [39]. Nagpal et al. showed that benzoic acid, potassium sorbate, and sodium nitrite each produced distinct compositional signatures with differential phylum-level and fermentative-taxa effects [49]. In vitro, de Souza Lopes et al. found that benzoate, sorbate, nitrite, and bisulfite each suppressed intestinal bacterial growth in a concentration-dependent manner, generally more potently than the NNS tested in parallel [44]. Hrncir et al. demonstrated synergy between fructose and preservatives on dysbiosis and metabolic dysfunction-associated steatotic liver disease in mice, underscoring dietary-context amplification [50], and Gerasimidis et al. found compound-specific effects of sodium benzoate and sodium sulfite on human fecal composition and fiber fermentation [15]. Preservatives are a medium-to-high priority class that warrants sub-classification by chemical family.

### 3.4. Colorants and Particulate Additives

Synthetic colorants and particulate additives fall into two interaction-mode subgroups. Synthetic azo dyes and other organic colorants (Allura Red AC E129, tartrazine E102, Sunset Yellow FCF E110) act mainly through host signaling and inflammatory cascades, with serotonin biosynthesis, a key mediator. Inorganic particulates (titanium dioxide, TiO_2_, E171; silicon dioxide, SiO_2_, E551) act through spatial and functional effects on the mucosal ecosystem—bacterial adherence, metabolite release, spatial ecology—without necessarily producing large shifts in alpha-diversity [40,43,51]; recognizing this prevents diversity metrics alone from under-detecting particulate effects. Kwon et al. showed in mice that chronic Allura Red AC at dietary-relevant doses exacerbates experimental colitis via upregulated intestinal serotonin, with both microbiota-dependent and independent components; fecal microbiota transfer from exposed donors worsened colitis in germ-free recipients, giving causal evidence for a microbiota-mediated component, and early-life exposure produced heightened, persistent susceptibility, which has direct pediatric risk-assessment relevance [40]. Granja-Iglesias et al. showed that both Allura Red and monosodium glutamate at dietary-relevant concentrations altered human fecal composition and affected intestinal epithelial function in co-culture [43]. For particulates, Rinninella et al.’s synthesis of 18 animal studies found that TiO_2_ consistently alters microbiota-associated functions and barrier integrity even without uniform compositional shifts [51], a dissociation that reinforces the need for multi-endpoint assessment.

The regulatory status of E171 is instructive. In 2021 the EFSA Panel on Food Additives and Flavorings concluded that E171 can no longer be considered safe as a food additive, principally because genotoxicity could not be ruled out [52], and the European Union banned its use in food from 2022. Other jurisdictions, including the United States, continue to permit it. This divergence is relevant to the review’s regulatory argument, but it should be read precisely. The EFSA conclusion rested on genotoxicity, specifically the inability to exclude it, and not on microbiome or barrier endpoints, which were reviewed but were not the basis of the opinion. The E171 case therefore does not show that microbiome-relevant concerns can drive regulatory change; it shows that an additive can lose its authorization on conventional toxicological grounds while a substantial microbiome and barrier literature sits alongside the decision without bearing on it, and that current frameworks can reach different conclusions from similar data. If anything the case illustrates the gap this review addresses rather than its closure, since the microbiome evidence for E171 was not in a form that could have contributed to the determination either way. This is a medium-priority class with emerging evidence; the two subgroups are treated separately below.

### 3.5. Thickeners, Stabilizers, and Bulking Agents

These mostly polysaccharide-based additives (xanthan gum E415, carrageenan E407, guar gum E412, pectin E440, maltodextrin, modified starches) modify texture, viscosity, and rheology. Their interaction modes vary: some act as selective microbial substrates depending on the community’s enzymatic repertoire; some affect mucosal biology through mucin structure or endoplasmic reticulum (ER) stress; some alter physical diffusion of nutrients and metabolites through rheological change [41,42]. Ostrowski et al. showed that xanthan-gum degradation is common in industrialized human microbiomes and is conferred by a few keystone degraders (including *Ruminococcus gnavus*) that initiate breakdown and enable cross-feeding; inter-individual differences in degrader abundance predict divergent ecological responses, and chronic dietary presence may enrich degraders over time [41]. Bellanco et al. found altered microbiota composition and intestinal homeostasis in mice exposed to carrageenan and noted explicitly that adding microbiome endpoints advances risk assessment, anticipating the framework below [42]. Gerasimidis et al. confirmed compound-specific effects of carrageenan and maltodextrin on human fecal abundance and fermentation [15]. Maltodextrin is separately notable for inducing ER stress and mucin depletion in epithelial cells, increasing mucosal vulnerability without direct antimicrobial action [13]. This is a medium-priority class with substantial mechanistic heterogeneity; the substrate-ecology (xanthan), mucosal-vulnerability (maltodextrin, carrageenan), and rheological pathways are developed below.

### 3.6. Flavor Enhancers

Flavor enhancers (monosodium glutamate, MSG, E621; disodium inosinate E631; disodium guanylate E627) are among the most consumed additives, used at high concentrations in savory processed foods and condiments. Proposed modes include altered bacterial amino-acid and neurotransmitter metabolism (glutamate is a GABA precursor), context-dependent ecological restructuring under dietary challenge, and indirect gut–brain effects via serotonin and GABA pathways [43,53,54]. The evidence base is thinner than for emulsifiers or NNS and points more consistently to context-dependence. Ahangari et al.’s systematic review found consistent MSG-associated microbiota perturbation across animal studies but marked heterogeneity in doses and backgrounds, with effects substantially stronger against high-fat or high-fructose backgrounds, supporting a reading of MSG as an amplifier of diet-induced dysbiosis rather than a potent disruptor in isolation [53]. Granja-Iglesias et al. showed that MSG at dietary-relevant concentrations altered community structure and epithelial function in human fecal co-culture, testing MSG and Allura Red in parallel to reveal additive-specific profiles [43]. Flavor enhancers are a low-to-medium priority class; despite limited mechanistic depth, high exposure, frequent co-occurrence with other microbiome-active additives, and gut–brain relevance mark MSG as a priority for future controlled human studies.

**Figure 1:**
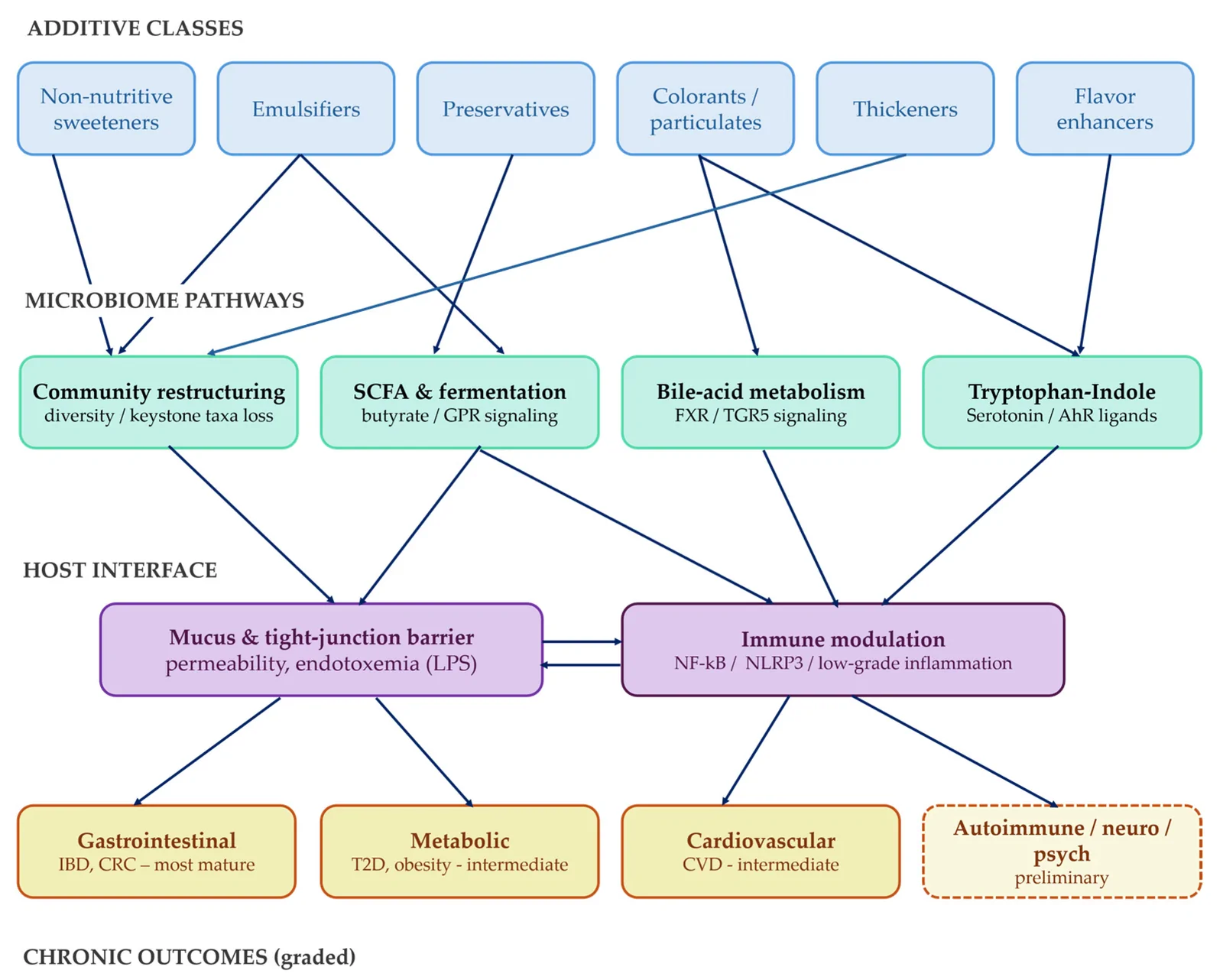
Convergent pathways from food additives to chronic-disease risk. Structurally diverse classes converge on four microbiome pathways, then on barrier and immune disruption, then on graded clinical outcomes. Baseline microbiome configuration modulates the direction and magnitude of response (direct evidence: sweeteners, emulsifiers). Solid boxes = graded support; dashed = preliminary.

Taken together, the six classes differ in mechanism, evidence tier, and the degree to which host susceptibility—particularly baseline microbiome configuration—shapes the direction and magnitude of response. This heterogeneity motivates the pathway-organized synthesis that follows, which integrates evidence from all six classes within shared frameworks (microbial ecology, metabolite production, barrier integrity, immune modulation), with implications for mixture effects and next-generation risk assessment. Figure 1 summarizes the four pathways and the classes that converge on each.

## 4. Mechanisms of Interaction Between Food Additives and the Gut Microbiota

Moving from association to mechanism requires an account organized by biology rather than by product category. This section identifies four interconnected primary mediators: alteration of microbial community structure and diversity; disruption of SCFA production and fermentative capacity; perturbation of bile acid metabolism; and disruption of tryptophan–indole pathways with neuroimmune consequences. Several classes converge on each pathway through distinct but overlapping mechanisms (Figure 1).

### 4.1. Alteration of Microbial Community Structure and Ecological Diversity

Compositional restructuring is the most consistently reported microbiome-level consequence of additive exposure. Emulsifiers are best characterized: the ex vivo screen by Naimi et al. showed that CMC, P80, lecithin, carrageenan, and polysorbate-20 each altered community composition and SCFA output, establishing ecological restructuring as a class-level property [7]; Panyod et al. confirmed compound-specific profiles in vivo, including altered *Firmicutes*: *Bacteroidetes* ratio and reduced *Akkermansia muciniphila* and *Faecalibacterium prausnitzii*, taxa with established roles in barrier integrity and immune tone [8]. NNS produce compound-specific changes strongly modulated by baseline configuration: Suez et al. found that saccharin, sucralose, aspartame, and stevia each produced distinct signatures whose direction and magnitude were partly predictable from baseline metagenomics, with baseline-stratified participants responding divergently to the same sweetener [11], a finding extended mechanistically by Rytter et al. for emulsifiers [10]. This convergence of NNS and emulsifiers on baseline configuration as a determinant of compositional response is, for those two classes, among the more consequential observations for risk assessment: population-average data under-specify the risk distribution, so stratified approaches are needed. Preservatives exert compositional effects through direct antimicrobial pressure [39,49], with reductions in *Bifidobacterium* and *Lactobacillus* and altered dominant *Firmicutes* among the more consistent changes, though compound specificity again argues against class-level generalization [15]. Whether this restructuring proceeds via surface-active interference (emulsifiers), altered substrate availability (NNS, thickeners), antimicrobial inhibition (preservatives), or serotonin-mediated immune remodeling (colorants), it is the upstream step from which the metabolic and immune disruptions below follow.

### 4.2. Disruption of Short-Chain Fatty Acid Production and Fermentative Capacity

SCFAs (acetate, propionate, butyrate) fuel colonic epithelial metabolism, help regulate tight-junction assembly, tune mucosal immune tone, and signal through G-protein-coupled receptors to influence systemic metabolism, including insulin secretion and adipogenesis. Because SCFA production depends on fiber-fermenting taxa (*F. prausnitzii*, *Roseburia* spp., *Bifidobacterium longum*), it is sensitive to compositional change. Gerasimidis et al. showed in human fecal batch cultures that several emulsifiers, sweeteners, and preservatives reduced overall SCFA output and altered acetate: propionate: butyrate proportions under standardized fiber-fermentation conditions, indicating that reduced fermentative capacity reflects functional alteration rather than merely reduced biomass [15]. Emulsifier exposure in mice reduces butyrate-producing taxa and caecal SCFAs alongside glucose intolerance and weight gain, consistent with reduced GPR41/GPR43 signaling and lower epithelial fuel availability [8]. Chi et al. link SCFA disruption to bile-acid signaling chronic sucralose inhibited farnesoid X receptor (FXR) signaling—a bile-acid-activated nuclear receptor that also modulates composition and SCFA signaling—providing a route by which NNS-induced changes cascade into lipid and cholesterol dysregulation [55]. The key translational limit is that direct colonic SCFA measurement in humans under controlled additive exposure remains scarce; Chassaing et al. reported metabolomic changes consistent with reduced fermentation but did not quantify SCFAs [9]. Future controlled studies should include validated fecal SCFA quantification (gas chromatography or NMR) as a functional endpoint linking compositional change to epithelial and metabolic outcomes.

### 4.3. Perturbation of Bile Acid Metabolism

Primary bile acids (cholic and chenodeoxycholic acid) are converted by microbial enzymes into secondary bile acids (deoxycholic, lithocholic, ursodeoxycholic acid) through deconjugation, dehydroxylation, and epimerization. The bile-acid pool acts as a host–microbiota co-metabolome, signaling through FXR, TGR5 (the Takeda G-protein-coupled receptor 5), and other receptors to regulate lipid and glucose metabolism, motility, and immunity. Because this biotransformation is exclusively microbial, any disruption of the microbiota can shift the pool toward a metabolically or inflammatorily dysregulated profile [55]. Chi et al. give the fullest characterization: chronic sucralose reduced the diversity of bile-acid-modifying taxa, shifted the pool toward a primary-acid-dominant profile, suppressed hepatic FXR target genes, and disrupted lipid and cholesterol homeostasis, effects that were attenuated when microbial diversity was preserved, confirming a microbiota-dependent pathway [55]. Emulsifier-induced restructuring likewise alters murine bile-acid profiles, with reduced secondary bile acids consistent with depletion of bile-salt-hydrolase-positive taxa [8]. Two qualifications govern how far this mechanism generalizes. The first concerns specificity. The FXR node is presented here not as a sucralose-specific lesion but as a candidate common downstream convergence point for any additive that depletes bile-salt-hydrolase-positive or SCFA-producing taxa, since deconjugation is the obligatory first step in secondary bile-acid formation and is carried by a functional guild rather than by a taxon. On that reading the sucralose data [55] and the emulsifier bile-acid shifts [8] are two instances of one pathway, and the prediction they share, which has not been tested directly, is that additives as chemically unlike as an emulsifier and a non-nutritive sweetener should converge on the same bile-acid signature to the extent that they deplete the same guild. The second qualification is that the pathway is not unidirectional, which complicates attribution. Butyrate itself modulates FXR signaling: butyrate supplementation reverses dysregulated bile-acid synthesis and the associated hepatic inflammation in FXR-deficient mice, in which butyrate-generating taxa and colonic butyrate are themselves reduced [56], and butyrate’s protective effect in enteric infection requires both bile-salt-hydrolase-producing flora and FXR, being abolished by inhibition of either [57]. Because SCFA loss and bile-acid disturbance are therefore reciprocally coupled, a community depleted of SCFA producers could either mimic the sucralose signature, through loss of butyrate-dependent FXR tone, or mask it, if FXR antagonism reduced by microbially modified bile acids offsets the primary-acid shift. Disentangling the two requires measuring SCFAs and the bile-acid pool in the same samples, with an FXR-pathway readout, rather than either alone. No controlled human trial has yet measured bile-acid pool composition as a primary outcome, and this should be built into next-generation designs, alongside paired SCFA quantification for the reason just given.

### 4.4. Disruption of Tryptophan–Indole Metabolic Pathways

Tryptophan enters three microbially mediated pathways: kynurenine (neuroactive compounds, NAD^+^ precursors), serotonin (roughly 90% of body serotonin via enterochromaffin 5-HTP synthesis), and indole (indole-3-propionic acid, indole-3-acetic acid, and other aryl-hydrocarbon-receptor [AhR] ligands with immune-regulatory properties). Their balance is set by the community’s enzymatic repertoire, and disruption is linked to inflammation, altered motility, immune dysregulation, and anxiety and depression [6]. Kwon et al. provide the most direct evidence of additive-induced disruption via serotonin: chronic Allura Red AC upregulated intestinal serotonin, promoting colitis susceptibility through altered mucosal immune tone and composition, with causal confirmation by microbiota transfer and pharmacological inhibition of serotonin synthesis [40]. The indole branch is affected by emulsifiers: as reviewed by Whelan et al., P80 reduces microbial indole production, decreases epithelial AhR signaling, and increases inflammation susceptibility, with reduced group-3 innate lymphoid cell (ILC3) activity and IL-22 production downstream [6]. Because indole-derived AhR ligands help maintain regulatory T cells and innate lymphoid cells at the mucosa, this pathway connects compositional change to the immune modulation described below. The three branches are interdependent, regulated partly by immune tone and by specific microbial populations, so additive-induced immune changes can feed back onto tryptophan metabolism and amplify an initial perturbation. This feedback is most relevant to chronic low-dose exposure, where small perturbations may compound over time; it is flagged as a plausible mechanism rather than a demonstrated one.

## 5. Intestinal Barrier Dysfunction

The intestinal barrier combines a luminal mucus gel, the epithelial monolayer with its tight-junction (TJ) network, the lamina propria immune compartment, and the underlying vasculature. Its integrity depends on coordinated mucin-secreting goblet cells, enterocytes, Paneth cells, and enteroendocrine cells, and is supported by microbial metabolites—butyrate, indole-propionic acid, secondary bile acids—that regulate TJ protein expression and epithelial renewal [6]. Additive-induced dysbiosis can therefore weaken the barrier by removing microbiota-derived support, on top of any direct additive effects on epithelial cells or mucus. Three interconnected mechanisms are set out below, and Table 4 summarizes the additive-barrier mechanisms by study model.

### 5.1. Mucus-Layer Disruption

The inner mucus layer, produced mainly by goblet-cell MUC2, is the primary spatial buffer between microbiota and epithelium. Surfactant-like emulsifiers have the strongest evidence for mucus disruption: both CMC and P80 reduce inner-mucus thickness, promote bacterial encroachment, and increase epithelial-adherent bacteria in mice, which are changes causally linked to low-grade inflammation and metabolic syndrome in germ-free reconstitution experiments [7,8,9]; and Naimi et al. showed that several emulsifiers alter the mucus-penetrating properties of resident bacteria ex vivo [7]. Gao et al. demonstrated synergistic mucus impairment under realistic co-exposure: carrageenan combined with a high-sugar diet synergistically impaired the colonic mucus barrier through microbiota-dependent mechanisms, including reduced *Akkermansia muciniphila* and decreased MUC2 [58]. This is directly relevant to mixture risk, since carrageenan and added sugars co-occur in most carrageenan-containing products, so isolated testing understates real-world effects. Maltodextrin gives a distinct mechanism: Zangara et al. showed that it impaired mucus-barrier function and accelerated colitis through direct epithelial effects, including goblet-cell ER stress, reduced MUC2 secretion, and increased susceptibility to luminal bacteria [13].

### 5.2. Tight-Junction Impairment

TJs (claudins, occludin, ZO-1/ZO-2, junctional adhesion molecules) regulate paracellular permeability; their assembly is governed by protein kinase C and myosin light-chain kinase signaling and supported by microbiota-derived butyrate (via histone deacetylase inhibition) and AhR ligands. NNS provide direct evidence for a host-receptor-mediated additive effect: Shil et al. showed that saccharin, sucralose, aspartame, acesulfame K, and stevioside disrupted Caco-2 barrier function through activation of the sweet-taste receptor T1R3, reducing transepithelial electrical resistance (TEER) and increasing paracellular permeability [59], a microbiota-independent mechanism that could compound microbiota-mediated impairment. In vivo, Nishimura et al. showed that dietary P80 impaired permeability with downstream disruption of skeletal-muscle glucose handling, illustrating multi-organ metabolic consequences [60]. In the controlled-feeding trial of CMC, the reported findings were reduced microbiota diversity, depletion of fecal short-chain fatty acids and free amino acids, and microbiota encroachment into the normally sterile inner mucus layer in two of seven exposed participants, the last of these being the barrier-relevant observation; fecal calprotectin did not change significantly [9]. The subsequent placebo-controlled trial of five emulsifiers likewise found no change in fecal calprotectin, *C*-reactive protein, or LBP, while lowering SCFA concentrations and increasing transcellular permeability for carrageenan [61]. Therefore, across the two human trials, the human barrier signal rests on encroachment and permeability rather than on a measurable inflammatory response at these durations. Across emulsifiers, NNS, and particulates, impaired TJ integrity is one of the most consistently reported cellular consequences [6]. Two limits apply to this mechanism, and the second is the more consequential. The strongest single-mechanism evidence here (Shil et al.) is a Caco-2 monolayer study, a Tier 1 model whose relevance to the intact human gut requires in vivo confirmation: T1R3 expression and function in the lower intestine in vivo are heterogeneous and need not reproduce those of a carcinoma-derived line. Beyond that, the mechanism is unlikely to be class-general, because the sweeteners tested differ in whether they reach the lower gut at all. Sucralose is the one compound for which the mechanism could plausibly operate distally: it is poorly absorbed, with more than 85% of an ingested dose reaching the colon unchanged, so luminal concentrations sufficient to engage epithelial T1R3 beyond the small intestine are credible [62,63]. The others are not. Acesulfame K is absorbed almost completely in the small intestine as the intact molecule and excreted in urine, leaving a colonic concentration that has been described as negligible; aspartame is hydrolyzed in the small-intestinal lumen and brush border to aspartate, phenylalanine and methanol, which are absorbed as such, so the intact molecule does not reach the colon at all; and saccharin is largely absorbed, with only about 15% of an ingested dose reaching the colonic lumen, so a colonic receptor-mediated effect would require intake well above habitual levels [62,63]. Steviol glycosides are a fourth case, passing the upper gastrointestinal tract intact but requiring bacterial hydrolysis to steviol before absorption. The Shil et al. finding should accordingly be read as compound-specific rather than as a property of non-nutritive sweeteners as a class: for sucralose it describes a mechanism that could plausibly operate on the colonic epithelium in vivo, whereas for acesulfame K, aspartame and saccharin the same in vitro observation is better interpreted as a receptor-level effect demonstrated at an anatomical site, and at concentrations, that habitual oral intake is unlikely to produce distally. T1R3 is also expressed proximally, so a small-intestinal effect is not excluded for the absorbed compounds; what the pharmacokinetics do not support is a colonic one. Any in vivo confirmation should be designed compound by compound, with luminal concentrations measured rather than assumed.

### 5.3. Increased Intestinal Permeability and Bacterial Translocation

The functional consequence of combined mucus and TJ disruption is increased paracellular permeability (FITC-dextran in animals; lactulose: mannitol in humans) and translocation of microbial components (lipopolysaccharide [LPS], bacterial DNA, peptidoglycan) or intact bacteria into the lamina propria and circulation. LPS translocation produces metabolic endotoxemia, associated with systemic low-grade inflammation, insulin resistance, and atherosclerotic risk, connecting barrier dysfunction to the chronic outcomes discussed below [6]. Gao et al. showed that carrageenan plus high sugar increased permeability alongside reduced claudin-1, occludin, and ZO-1, demonstrating that nutritionally realistic co-exposures, not only pharmacological doses, produce functionally meaningful compromise [58]. In humans, circulating permeability markers (fatty-acid-binding protein-2 [FABP2], lipopolysaccharide-binding protein [LBP]) have been associated with artificial-sweetener intake in observational studies, consistent with low-level barrier impairment under habitual consumption, though direction of effect cannot be established cross-sectionally [6]. Integrating functional permeability endpoints, not only compositional metrics, into additive safety evaluation is a specific recommendation below.

## 6. Immune Modulation

Additive-induced changes in microbial composition, barrier integrity, and metabolite output converge on the mucosal immune system. Three axes—chronic low-grade inflammation, pro-inflammatory signaling, and immune–metabolic consequences—are interconnected and often co-activated [6,54].

### 6.1. Low-Grade Chronic Inflammation

A recurring feature across models is the induction of low-grade, chronic inflammatory tone: mild elevations in TNF-α, IL-6, and IL-1β, modest sustained macrophage activation, and subclinical permeability increases. This matters because it operates below the clinical threshold while progressively affecting metabolic and vascular tissue [6]. In the CMC feeding trial a subset of participants developed microbiota encroachment into the inner mucus layer without symptoms, although fecal calprotectin did not change significantly, so the trial shows a spatial barrier change rather than a measurable inflammatory response at eleven days [9]. Kwon et al. showed that chronic Allura Red AC raised intestinal serotonin, sustaining a low-grade inflammatory state and heightening colitis susceptibility, with early-life exposure producing persistent sensitization after withdrawal [40]. Rinninella et al.’s synthesis found that TiO_2_ consistently elevates pro-inflammatory markers and alters barrier-associated immune function even without overt colitis [51]. Human evidence for additive-induced low-grade inflammation remains limited to a few controlled trials and case–control studies, and inflammatory biomarkers have not been systematically built into human exposure studies.

### 6.2. Pro-Inflammatory Signaling Pathways

Additive-induced inflammation converges on a small set of signaling nodes: NF-κB activation, NLRP3 inflammasome assembly (maturation of IL-1β and IL-18), and Toll-like receptor (TLR) engagement by translocated microbial components [6]. Emulsifier-induced barrier disruption allows TLR4 engagement by translocated LPS, activating NF-κB in lamina propria macrophages and dendritic cells and sustaining cytokine production in a positive-feedback loop. Gao et al. showed that carrageenan plus high sugar produced simultaneous NF-κB activation and elevated colonic TNF-α and IL-6, illustrating barrier-mediated activation by realistic co-exposures [58]. Inflammasome activation appears across classes: NNS-induced dysbiosis has been associated with NLRP3 activation in epithelial cells via altered metabolites and oxidative stress, and sucralose can modulate T-cell activation and cytokine production partly independently of the microbiota, indicating direct adaptive-immune effects [6]. The emerging picture is convergent innate-immune activation through complementary routes—barrier disruption and LPS translocation (emulsifiers), T1R3-mediated epithelial signaling (NNS), serotonin-driven sensitization (colorants), and antimicrobial dysbiosis (preservatives). This raises the possibility that simultaneous exposure could produce synergistic rather than merely additive immune activation. That synergy is, so far, demonstrated directly only for the carrageenan–sugar combination [58]; for other combinations it remains a hypothesis, now partly testable against the additive-mixture epidemiology discussed below.

### 6.3. Immune–Metabolic Consequences

The interface between gut immune activation and systemic metabolic dysfunction is among the most clinically consequential pathways. Metabolic endotoxemia—subclinical circulating LPS from barrier disruption—activates hepatic and adipose inflammatory signaling, driving insulin resistance, adipose inflammation, and dyslipidemia via TLR4 on peripheral immune cells and hepatocytes [6]. Murine CMC and P80 studies document not only intestinal inflammation but weight gain, hepatic steatosis, and hyperglycemia downstream of sustained low-grade endotoxemia [8,9]. These mechanisms align with the prospective cohort evidence below: emulsifier exposure has been associated prospectively with increased cardiovascular risk, consistent with endotoxemia-driven vascular inflammation [64], and the NNS-cardiovascular association is plausible through the sucralose–FXR–bile-acid pathway and impaired SCFA-mediated anti-inflammatory signaling [65]. Seto et al. provide a broader synthesis of additive-induced gut disruption propagating to systemic immune-metabolic outcomes [54]. These converging lines motivate including immune-relevant endpoints—serum cytokines, LBP, FABP2, fecal calprotectin—alongside compositional metrics in next-generation evaluation.

## 7. Gut–Brain Axis Alterations

The gut–brain axis (GBA) integrates neural (vagus nerve, enteric nervous system), endocrine (hypothalamic–pituitary–adrenal [HPA] axis, gut hormones), and immune pathways that link gut microbial ecology to central nervous system function, affect, cognition, and behavior. Microbial metabolites—SCFAs, indole derivatives, secondary bile acids, neurotransmitter precursors—mediate this interface, and their disruption by additives gives a plausible basis for the additive-associated neurological and psychiatric effects seen in experimental models [6,66,67,68]. It must be stressed at the outset that the human evidence here is thin and that most findings are animal-derived, often at doses above typical human exposure.

### 7.1. Neurotransmitter Alterations

The best-characterized evidence concerns aspartame’s effects on glutamatergic and GABAergic signaling. Jones et al. showed that chronic aspartame at doses equivalent to 8–15% of the FDA maximum daily intake induced anxiety-like behavior with altered glutamate-GABA ratios and amygdala gene expression in mice and reported that the phenotype was transmissible across at least two generations through apparent transgenerational epigenetic mechanisms [66]. This is a striking finding, but it rests on a single rodent model; extrapolation to human pregnancy or childhood risk is not warranted and is presented here as a hypothesis that motivates controlled study, not as an established risk. The proposed mechanism involves aspartame-derived phenylalanine and aspartate competing with tryptophan for large-neutral-amino-acid transporter access, potentially reducing serotonin synthesis while raising glutamatergic tone [6]. The serotonin pathway is independently implicated by colorants: Kwon et al. showed that Allura Red AC raises intestinal serotonin via enterochromaffin stimulation, altering motility and mucosal immune tone [40]; because most body serotonin is produced in the gut and reaches the GBA via vagal afferents and platelet transport, elevated gut serotonin could plausibly influence central serotonergic tone, again a hypothesis requiring controlled evaluation. Acesulfame K has also been implicated in microbiota-mediated anxiety-like behavior in rodents [6].

### 7.2. Stress Response and Neuroendocrine Modulation

The HPA axis is bidirectionally coupled to the microbiota through vagal afferent signaling, microbial SCFA sensing, and cytokine-mediated communication. Butyrate regulates HPA reactivity through epigenetic effects on hippocampal glucocorticoid-receptor expression, giving a basis for additive-induced microbiota changes to alter stress reactivity [68]. Kurhaluk et al. reviewed the integrative roles of the parasympathetic nervous system, vagus nerve, and microbiota, concluding that the vagal afferent pathway is a primary route by which microbiota-derived signals influence neuroendocrine responses, and that dysbiosis, including additive-induced dysbiosis, can dysregulate HPA reactivity [68]. Early murine studies showed that dietary CMC and P80 induced anxious behavior alongside intestinal inflammation, suggesting that microbiota-mediated inflammatory signaling can reach the CNS via vagal or cytokine pathways [6].

### 7.3. Cognitive and Behavioral Effects

Cognitive and behavioral effects are documented in animal models but poorly characterized in humans. Jones et al. showed that aspartame impaired learning and memory in mice, with reduced hippocampal neurotrophin signaling and prefrontal gene expression consistent with neuroinflammation [66]. Zebrafish exposed to MSG and tartrazine, singly and in combination, over 30 days showed reduced social interaction and object preference, increased anxiety-like behavior in the combined-exposure group, altered acetylcholinesterase activity with disturbed serotonin and cortisol levels, and gut microbiota change including increased Enterobacter, with the gut–brain axis proposed as mediator [69]; the exposure concentrations in that study were in the thousands of parts per million and far above dietary intake, so it establishes a pathway rather than a dietary risk; tartrazine in rodents produced similar neurotransmitter imbalances alongside microbiota changes, suggesting that colorants may act through both direct (neurotoxic metabolite) and indirect (microbiota-mediated) routes. Goel et al. synthesize the microbiota-GBA pathway in anxiety neurobiology, identifying the same mediators (GABA, serotonin, indole derivatives, HPA regulation) implicated in additive-associated perturbation [67]. The dominant caveat is that this evidence is predominantly animal-derived, often at doses exceeding human exposure, with human data on additive-specific cognitive or behavioral effects currently lacking, a high-priority gap given the public-health significance of chronic neurological effects of ubiquitous additives, particularly during developmental windows.

## 8. Association with Chronic Diseases: Graded Evidence Across Six Domains

The mechanistic pathways described above—compositional dysbiosis, SCFA and metabolite impairment, barrier dysfunction, immune activation, and GBA perturbation—form a biologically coherent cascade through which additive exposure could contribute to chronic disease. That the cascade is internally coherent raises its plausibility; it does not establish that the pathway operates at habitual dietary doses, or that it accounts for the modest associations reported below. The evidence is graded across six domains along the three pre-specified anchored dimensions (Table 5). Two points frame what follows. First, the grading describes evidentiary maturity, not a license for action: even the best-supported domains rest on surrogate-endpoint human trials and single-cohort epidemiology. Second, the effect sizes in the underlying prospective studies are modest, and they are reported so that statistical significance is not mistaken for large biological effect. Because these are relative measures, their public-health weight depends on the absolute risk difference across the realistic exposure range and on the population attributable fraction, neither of which the source analyses estimated; a hazard ratio close to unity per small intake increment can correspond to a negligible absolute effect. The direction of effect across the six domains is summarized in Table 6.

### 8.1. Gastrointestinal Diseases

Gastrointestinal diseases—inflammatory bowel disease (IBD: Crohn’s disease, ulcerative colitis), colorectal cancer (CRC), irritable bowel syndrome—have the strongest and most methodologically diverse evidence. The mechanistic basis rests on barrier disruption and immune activation: emulsifier-induced mucus thinning, TJ impairment, bacterial encroachment, and low-grade inflammation give a coherent route from consumption to IBD exacerbation. Whelan et al. conclude that additives, particularly emulsifiers and carrageenan, are among the more biologically plausible dietary contributors to IBD activity [6]. Katsoudas et al. found dietary emulsifier exposure substantially higher in people with IBD than in controls, with several consumed above levels that produce IBD-like phenotypes in animals [37]; as a case–control comparison, this is open to reverse causation, because diagnosis and symptoms can themselves alter dietary choices. Soublette Figuera et al.’s scoping review reported that additive-excluding diets improved IBD disease activity [38]; because such diets remove many co-occurring components of ultra-processed foods at once, this supports a role for the dietary pattern but does not isolate any single additive. CRC risk has been linked to emulsifier intake through microbiota-mediated secondary-bile-acid production and chronic inflammation [21], and Choi and Moon identify additives as plausible mediators of the UPF–IBD association, given that UPF nutritional composition alone does not account for the signal [73]. Grading: association Moderate (the evidence does not include two independent prospective cohorts of additive intake with incident disease, as the Strong anchor requires, and the three conditions grouped in this domain differ substantially, as set out in Table 5, notes 2 and 3); mechanistic plausibility Strong; causal confidence Moderate, limited by the absence of large RCTs with incident disease as a primary endpoint and by the non-additive-specificity of the dietary-exclusion evidence. This is the one domain where the association rests on more than a single cohort, combining case–control, dietary-exclusion, and mechanistic lines. The non-specificity of the dietary-exclusion evidence is not an incidental gap but a consequence of what can feasibly be randomized, and it is worth being explicit about why. A trial that isolated one additive’s effect on IBD incidence would need to randomize healthy participants to long-term exposure at habitual intake and follow them to a first diagnosis. Given the low population incidence of inflammatory bowel disease and a plausible hazard ratio near those seen for other additive-outcome pairs, the required sample size and duration are outside what is fundable, and deliberately feeding a suspected pro-inflammatory compound for years to prevent nothing is not defensible ethically; blinding is a further obstacle, since emulsifiers and thickeners carry the texture of the foods that deliver them. Trials in established disease are more tractable but answer a narrower question. Even there, the realistic design is subtractive rather than additive: withdrawing additives from the diet of patients in remission and measuring time to relapse, which is what the exclusion studies approximate but without isolating a compound. Single-additive supplementation on top of an additive-free background is the design that does isolate a compound, and it has now been run in healthy volunteers at a scale that shows both its feasibility and its limits: five emulsifiers administered for four weeks against placebo lowered SCFA concentrations, increased transcellular permeability for carrageenan, and left fecal calprotectin, *C*-reactive protein, LBP, and the serum inflammatory proteome unchanged [61]. A trial of that structure in quiescent IBD, powered for relapse or for endoscopic activity rather than for biomarkers, stratified by baseline microbiome configuration, and of a duration measured in months rather than weeks, is the feasible decisive study. Incidence is not a realistic primary endpoint for any additive, and the honest implication is that causal attribution for IBD incidence will have to rest on triangulation across cohorts, mechanism, and disease-activity trials rather than on a single definitive RCT.

### 8.2. Metabolic Disorders

Type 2 diabetes (T2D) and obesity-associated metabolic syndrome have the second-highest evidentiary maturity, but the epidemiology is concentrated in one cohort. The prospective NutriNet-Santé analysis by Salame et al. (104,139 participants, 1056 incident T2D cases, mean 6.8 years) reported positive associations between several emulsifiers and incident T2D, with modest per-increment hazard ratios: total carrageenans (E407) 1.03 (95% CI 1.01–1.05) per 100 mg/day, tripotassium phosphate (E340) 1.15 (1.02–1.31) per 500 mg/day, guar gum (E412) 1.11 (1.06–1.17) per 500 mg/day, and xanthan gum (E415) 1.08 (1.02–1.14) per 500 mg/day [70]. The carrageenan estimate here refers to total carrageenan as captured by food-composition databases and does not resolve the molecular-weight fractions distinguished earlier. NNS have been prospectively linked to T2D in the same cohort, with sucralose and acesulfame K strongest [71], plausible through the Suez RCT (microbiome-dependent glycemic responses) [11] and the Chi sucralose-FXR mechanism [55]. The recent extension to additive mixtures is relevant because it brings the co-exposure question to population data, although it does not test it experimentally: in the same cohort (108,643 participants, 1131 T2D cases), a mixture dominated by modified starches, pectin, gums, carrageenan, and polyphosphates was associated with incident T2D (HR 1.08, 95% CI 1.02–1.15 per 1 SD), as was a mixture characteristic of artificially sweetened drinks that included acesulfame K, aspartame, and sucralose (HR 1.13, 95% CI 1.08–1.18 per 1 SD) [36]. Several cautions temper this. The per-increment effects are small, and the entire prospective signal for metabolic disease comes from a single volunteer cohort with indirect additive-exposure estimation. These analyses also test many additives and, for the mixtures, use data-driven exposure definitions, so multiple-comparison and model-selection flexibility should temper interpretation; and because a data-driven mixture of co-occurring additives partly re-captures the overall ultra-processed dietary pattern, a mixture association is weaker evidence of an additive-specific co-exposure effect than of the pattern itself. Replacing caloric sweeteners with NNS may not be metabolically neutral for susceptible individuals, but the population-level effect estimates are modest and not yet replicated in an independent cohort. Grading: association Moderate (down-graded from a naïve reading precisely because the ≥2-cohort anchor is not met); mechanistic plausibility Strong; causal confidence Moderate, resting on glycemic-surrogate RCT evidence [11] rather than disease-endpoint trials.

### 8.3. Cardiovascular Disease

Cardiovascular disease (CVD) evidence has grown rapidly since 2022 but shares the single-cohort limitation. Sellem et al. reported prospective associations between emulsifier intake and increased CVD in NutriNet-Santé (95,442 adults; 1995 CVD events), with total celluloses at HR 1.05 (95% CI 1.02–1.09) and mono-/diglycerides (E471/E472) at HR 1.07 (1.04–1.11) for total CVD, and comparable small associations for coronary and cerebrovascular endpoints [64]. Debras et al. found artificial-sweetener intake (mainly aspartame, acesulfame K, sucralose) positively associated with CVD in the same cohort [65], and a further NutriNet-Santé analysis reported that preservative additives, including nitrites, were associated with a higher incidence of hypertension and cardiovascular disease [72]. Plausibility is supported by the immune-metabolic cascade described above: endotoxemia from barrier disruption activates TLR4 in vascular endothelium and macrophages, promoting atherosclerosis through NF-κB-driven cytokine production. Warner and Khan argue that consistent UPF–CVD evidence is not adequately reflected in current regulation and that additives are likely contributors [74]. Grading: association Moderate; mechanistic plausibility Moderate; causal confidence Low, with no RCT evidence for CVD endpoints and unresolved dietary-pattern confounding.

### 8.4. Autoimmune Diseases

Autoimmune diseases—rheumatoid arthritis, systemic lupus erythematosus, inflammatory arthritis, multiple sclerosis—have plausible mechanistic involvement but limited, preliminary direct epidemiological evidence. The basis lies in the immune modulation described above: additive-induced dysbiosis and barrier impairment could promote molecular mimicry, reduce regulatory-T-cell generation through impaired SCFA and AhR-ligand availability, and sustain systemic pro-inflammatory cytokines that lower the threshold for autoimmune tissue damage [6]. Recent cohort analyses have begun reporting associations between higher UPF intake and autoimmune disease risk, though additive-specific attribution is not yet delineated [6]. Allura Red AC alters regulatory immune-cell trafficking [40], and NNS can modulate T-cell function partly independently of the microbiota [6]; if sustained, such effects could contribute to systemic autoimmune sensitization. However, the causal pathway involves several steps (barrier disruption, immune priming, loss of peripheral tolerance) studied largely in isolation. Grading: association Weak; mechanistic plausibility Moderate; causal confidence Low, with no interventional evidence and substantial confounding.

### 8.5. Neurological and Psychiatric Disorders

Neurological (cognitive decline, dementia, Parkinson’s disease, ADHD) and psychiatric (depression, anxiety, autism spectrum) conditions have the most preliminary evidence. Plausibility derives from the gut–brain-axis pathways described above. Large cohorts link higher UPF consumption to accelerated cognitive decline and increased depression risk; additive-specific attribution has not been established, though additive content is a plausible contributor [6]. The experimental evidence—the aspartame–anxiety work of Jones et al. [66], the colorant-serotonin pathway of Kwon et al. [40], and the MSG/tartrazine gut–brain evidence of Vaithilingam et al. [69]—is predominantly animal-derived at doses that, in the zebrafish study explicitly, exceed human dietary exposure by orders of magnitude. ADHD has been associated with artificial-colorant exposure (tartrazine, Allura Red) in pre-microbiome-era RCTs in children, but microbiota-mediated mechanisms have only recently begun to be characterized [6]. Grading: association Weak; mechanistic plausibility Emerging; causal confidence Low, resting predominantly on animal evidence with human disease-endpoint data lacking.

## 9. Limitations of the Current Evidence Base and Translational Challenges

Several structural limitations constrain the inferences currently possible; they also define the research priorities needed to move from mechanistic plausibility to causal confidence.

Dependence on a single epidemiological cohort. The prospective additive-specific signal for metabolic and cardiovascular disease comes almost entirely from NutriNet-Santé [36,64,65,70,71]. This is a large, well-characterized cohort, but it is one cohort, and it carries features that bound interpretation: participants are self-selected volunteers, predominantly women and more highly educated than the general population, which limits generalizability and can bias associations in either direction; and additive exposure is estimated indirectly, by matching reported foods to composition and brand databases rather than by measurement, introducing substantial exposure misclassification that generally, though not invariably, attenuates associations toward the null. The effect estimates are also modest (per-increment hazard ratios typically 1.03–1.15). The metabolic and cardiovascular domains have been graded to reflect these facts rather than reading consistency within one cohort as independent replication. Because the type 2 diabetes, cardiovascular and mixture analyses share that cohort, exposure method and confounding structure, their agreement is not the mutual corroboration that independent cohorts would provide. A formal ROBINS-E appraisal of the pivotal NutriNet-Santé analyses (Table S2) places them at high overall risk of bias, driven by that exposure-measurement domain. This is the principal reason the corresponding GRADE certainty (Table 2) does not exceed low for the metabolic and cardiovascular outcomes. Confirmation in cohorts with different populations and, ideally, biomarker-based exposure assessment is the single most valuable epidemiological advance the field could make.

Dose realism and exposure characterization. A persistent limitation is the use of exposure concentrations above realistic human intake. The CMC/P80 studies of Chassaing et al. and the multi-emulsifier study of Panyod et al. used broadly realistic doses, but many mechanistic studies—particularly for preservatives, colorants, and particulates—use doses one to two orders of magnitude above dietary levels [6,8]. Because ADIs incorporate safety margins built on realistic exposure, supra-physiological findings cannot be extrapolated to human risk without explicit dose–response characterization and exposure-realistic replication. Table 7 sets the tested doses of the principal studies against ADIs and estimated intakes where these are available and marks the compounds for which the comparison cannot yet be made. Future designs should anchor concentrations to population dietary-survey data, including 95th-percentile high-exposure estimates.

Mixture exposure and co-occurrence. A single UPF product may contain five to fifteen additives from several classes. The mechanistic evidence identifies several classes converging on shared pathways—barrier disruption (emulsifiers, carrageenan), SCFA impairment (emulsifiers, NNS, preservatives), immune activation (emulsifiers, colorants, particulates)—under which mixtures could produce additive or synergistic effects even when individual exposures are sub-threshold. Direct evidence is still limited: the carrageenan–sugar synergy of Gao et al. remains the main experimental proof of concept at realistic co-exposure levels [58], with the synergy between fructose and preservatives reported by Hrncir et al. as a second instance [50], and the additive-mixture epidemiology from NutriNet-Santé [36] is associational and from the same single cohort. Broad claims of mixture synergy are therefore not yet warranted, and the design requirements for testing them rather than inferring them are set out in Section 10.3. Current EFSA and FDA procedures assess individual additives against ADIs without systematic consideration of co-occurrence or pathway convergence.

Evidence-tier constraints and translation gaps. The five-tier hierarchy imposes explicit inference limits. Animal models—the most prevalent tier—differ from humans in microbiota composition, intestinal architecture, dietary background, and life-course exposure, limiting extrapolation of dose–response and magnitude. Germ-free and antibiotic-attenuation designs that provide causal proof of microbiota dependency are not feasible in humans, so human causal evidence relies on controlled feeding trials with surrogate endpoints. Such trials are further constrained by duration (usually 2–4 weeks), scale (underpowered for modest effects in heterogeneous populations), and endpoint selection (rarely including functional microbiome or barrier assays alongside compositional metrics).

Host susceptibility and the risk of an unfalsifiable thesis. For NNS and emulsifiers, individual responses are partly predictable from baseline composition [10,11], so populations with different baseline distributions will show different average effects under identical exposure. Population-average estimates therefore underspecify the risk distribution. One tempting move is, however, deliberately avoided: explaining away every discordant or null result as “susceptibility heterogeneity.” Used indiscriminately, that framing is unfalsifiable. It should be invoked only where baseline-stratified data exist; where they do not, a null result—such as the within-ADI aspartame/sucralose trial that found no compositional change [12]—should be allowed to count as evidence against an effect.

Reliance on secondary sources. Parts of the mechanistic synthesis, especially for the indole-AhR-ILC3 axis and some immune and neurological pathways, rest on a small number of authoritative reviews rather than primary studies. Claims have been attributed to primary sources wherever possible, and review-level support has been flagged explicitly (for example, “as reviewed by Whelan et al. [6]”); readers should treat those statements as syntheses of others’ primary data, not as independent primary evidence. Primary studies have been used in place of review-level support wherever one was available for the specific claim. The indole-AhR-ILC3 axis remains the one pathway where this review still relies principally on review-level synthesis, and the claims made about it are correspondingly weak. More broadly, the mechanistic literature probably over-represents positive findings: studies reporting additive-induced dysbiosis are more readily published than null screens, preprints were not searched, and the GRADE assessments did not apply a separate downgrade for publication bias, so the mechanistic evidence base should be read with that asymmetry in mind.

## 10. An Operational Framework for Microbiome-Inclusive Food Additive Risk Assessment

**Figure 2:**
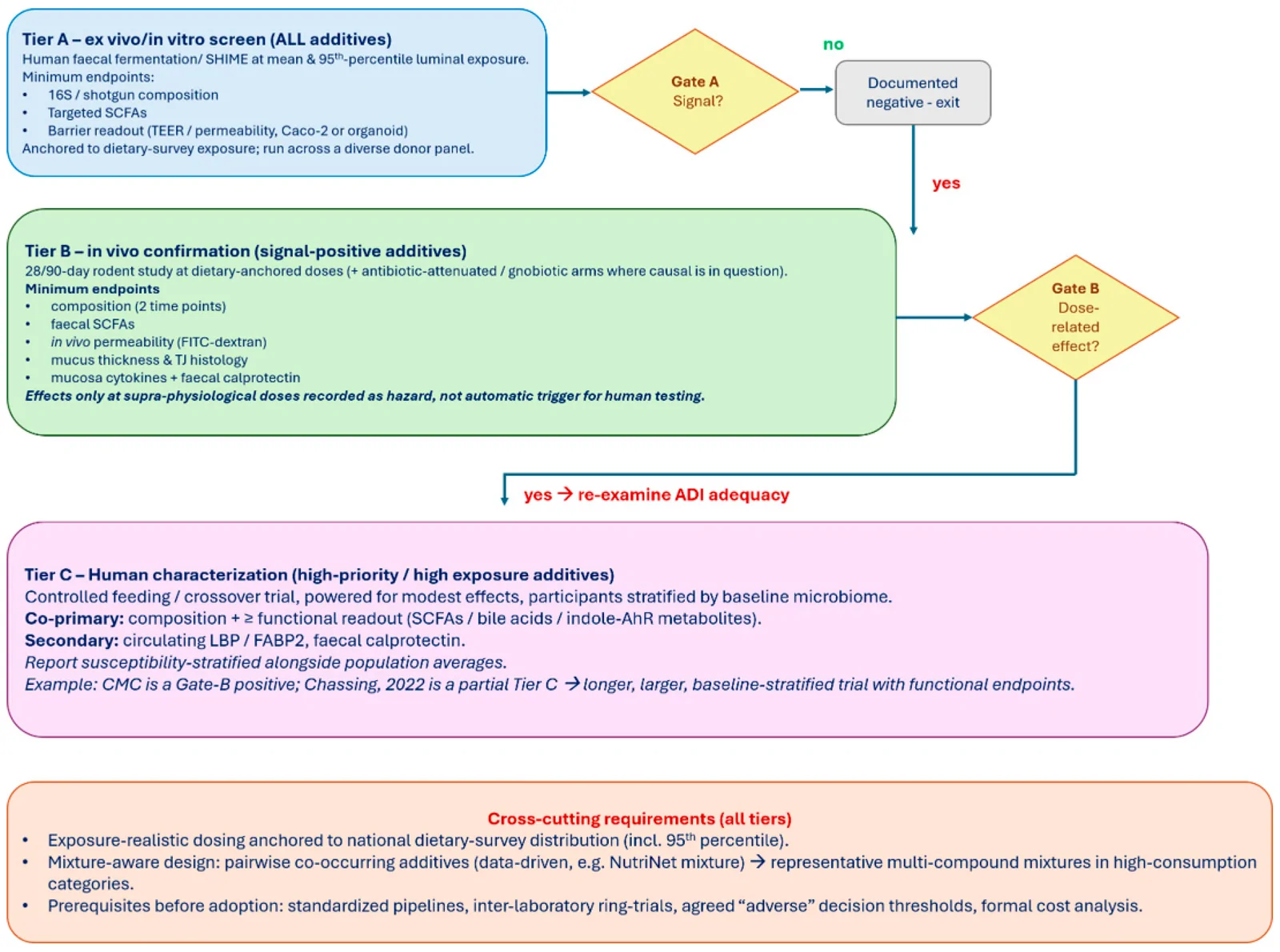
Tiered, decision-gated protocol for microbiome-inclusive additive evaluation. Gates A and B are intended to resolve additives at the lowest adequate tier, with particulates and nanomaterials entering at Tier B; the resource implications have not been quantified. The framework operationalizes the EFSA GI-microbiome roadmap [22] for additives. The mixture example shown in the figure is the NutriNet-Sante additive-mixture analysis [36]; the partial Tier C example is the carboxymethylcellulose controlled-feeding trial [9].

The evidence points to one normative conclusion: the biology of additive–microbiota interactions is not captured by current additive-evaluation practice, and the tools to capture it now exist. EFSA has itself set out a roadmap for integrating gastrointestinal microbiomes into risk assessment [22] and guidance on characterizing microorganisms in the food chain [23]. What is missing is not the principle but an additive-specific, decision-gated procedure. This section provides one. It is designed to sit inside existing regulatory-toxicology infrastructure (OECD subchronic and chronic study designs; EFSA re-evaluation workflows) rather than to require new institutions, and it is deliberately tiered so that most additives are resolved at the earliest adequate tier, with human studies reserved for compounds that trigger them. Figure 2 presents the framework as a decision tree. The text below specifies the tiers, endpoints, and pass/fail gates.

### 10.1. The Tiered Decision Procedure

Tier A—Ex vivo/in vitro screen (all additives). Every additive entering evaluation or re-evaluation is screened in human fecal batch fermentation or a validated dynamic model (for example SHIME), at concentrations anchored to estimated luminal exposure at mean and 95th-percentile intake. Minimum endpoints: 16S or shotgun compositional profiling, targeted SCFA quantification, and a barrier-relevant readout in a paired epithelial model (TEER or FITC-permeability in Caco-2 or organoid monolayers).

Donor panel. Donors are run individually rather than pooled, because pooling averages away the between-donor heterogeneity Tier A exists to detect. Minimum twelve unrelated adult donors, selected to span rather than to represent the compositional range: at least three per dominant enterotype (*Bacteroides*-, *Prevotella*- and *Ruminococcaceae*-dominated) and at least two low-richness, low-cell-count profiles of the *Bacteroides*-2 type, these being the configurations associated with weaker colonization resistance and with discordant responses in fecal-transfer trials [77]. Donors are additionally characterized for the functional guilds through which the known mechanisms run: bile-salt-hydrolase carriage, butyrate-synthesis capacity (but/buk genes) and mucin-degrading taxa. For emulsifiers, baseline metagenomic signatures predicting CMC sensitivity already exist and could be used to enrich the panel [10]; parallel small-volume reactor arrays make a panel of this size tractable with documented between-run reproducibility [78].

Gate A thresholds. An additive is signal-positive if, at the concentration equivalent to 95th-percentile intake, any one of the following holds in at least three of twelve donors and is reproduced in an independent run: a compositional shift exceeding the assay’s donor-specific technical variance against a pre-registered threshold rather than a nominal *p*-value; a reduction in total or butyrate SCFA concentration of at least 20% against vehicle-matched control; or a fall in TEER of at least 15%, or the corresponding rise in paracellular tracer flux, in the paired epithelial model. Tier A is powered to these effect sizes a priori, and the pre-registered thresholds rather than post-hoc significance determine the outcome. These three values are indicative rather than validated: they are set to sit above typical inter-run variation while remaining below the effect sizes reported for the best-evidenced emulsifiers, and calibrating them against inter-laboratory data is a prerequisite rather than a detail (Text S3).

Gate A exit. An additive producing none of these signals exits with a documented negative, subject to two restrictions. A Tier A negative is a negative for microbiota-mediated and microbiota-proximal barrier effects only; it is not a clearance and does not displace the conventional toxicological endpoints running in parallel. The exit is also unavailable to compounds whose principal mechanism a cell-free fermentation vessel cannot express: particulates and nanomaterials, including any additive with a measurable nanoscale fraction, act substantially through uptake into gut-associated lymphoid tissue—for which a fermentation reactor has no Peyer’s patches, no dendritic or M cells and no translocation route—and these bypass Gate A and enter at Tier B by default.

Section 10.5 shows what happens when this exemption is absent. The rationale for each threshold and for the panel composition is given in Text S3. An additive that produces a signal, or that falls within the exemption, proceeds to Tier B.

Tier B—In vivo confirmation (signal-positive additives). A 28 or 90-day rodent study (conventional plus, where mechanism is in question, antibiotic-attenuated or gnotobiotic arms, for which the methodological constraints are well described [35]) at dietary-anchored doses. Minimum endpoints: compositional profiling at two time points; fecal SCFAs; in vivo permeability (FITC-dextran); histological mucus thickness and TJ-protein distribution; and mucosal cytokines plus fecal calprotectin.

Gate B thresholds. Three conditions must be met jointly. Consistency: the same direction of effect in at least two dose groups, or in two independent cohorts or laboratories, with both sexes represented and analyzed separately, since several additives show sex-discordant effects that a pooled analysis conceals. Dose-relation: a monotonic trend across at least three dose levels spanning estimated mean intake to the ADI, tested as a trend rather than by pairwise comparison against control. Adversity: at least one host endpoint, not composition alone, must move in the direction of harm. This last condition carries most of the weight, because it separates an adaptive microbiome change from an adverse one. Compositional change on its own is not an adverse effect; it is read as adverse when accompanied by loss of a functional output (SCFA or bile-acid pool), by structural or functional barrier compromise (mucus thickness, tracer permeability, tight-junction distribution), or by mucosal immune activation, and when it does not revert during a recovery period after exposure ends. A reversible compositional shift without functional or host correlate is recorded as a biological response, not a hazard. Where composition and host outcome dissociate, a microbiota-dependence arm (antibiotic-attenuated, germ-free or fecal-transfer) establishes whether the microbiota is necessary, sufficient or neither; this arm is diagnostic rather than optional, since a host effect that does not transfer with the microbiota is real but is not a microbiome-mediated effect and is routed to conventional immunotoxicological assessment instead. Effects seen only at supra-physiological doses are recorded as hazard information without automatically driving human testing. Effects meeting all three conditions trigger Tier C and inform whether the existing ADI adequately covers microbiome-mediated effects.

Tier C—Human characterization (high-priority or high-exposure additives). A controlled feeding or crossover trial, powered for the modest effect sizes the field sees, with participants stratified by baseline microbiome configuration. Co-primary endpoints: compositional change and at least one functional readout (SCFAs, bile-acid profile, or indole/AhR-ligand metabolites); secondary endpoints: circulating LBP/FABP2 and fecal calprotectin. Trials should report susceptibility-stratified estimates alongside population averages.

### 10.2. Minimum Endpoint Set and Why Each Is Included

The framework specifies endpoints rather than leaving “microbiome assessment” undefined. Composition (16S minimum, shotgun preferred) detects restructuring but, on its own, misses functionally important changes; particulate TiO_2_ and some NNS alter function with little compositional shift [6,51], so compositional endpoints alone risk false negatives. Functional endpoints—targeted SCFAs, secondary bile acids, tryptophan/indole metabolites—are therefore co-primary, not optional. Barrier endpoints (TJ proteins, in vivo permeability, circulating FABP2/LBP/zonulin) capture the mechanistic gateway from dysbiosis to systemic effects. Immune endpoints (mucosal cytokines, fecal calprotectin) capture the low-grade inflammation that symptom-based monitoring misses [9]. Table 8 maps each pathway to its recommended endpoint and model.

### 10.3. Exposure-Realistic and Mixture-Aware Design

Two requirements cut across all tiers. They are stated here as design requirements because both are more demanding than they appear. Preclinical concentrations must be anchored to population dietary-exposure distributions from nationally representative survey data, not chosen for effect. Mixture testing should be incorporated through a tractable escalation: begin with pairwise combinations of co-occurring additives identified from dietary-survey and product-formulation data (the NutriNet-Santé mixtures [36] are a data-driven starting point) and escalate to representative multi-compound mixtures only in high-consumption product categories. This improves realism without an unmanageable combinatorial burden. Detecting interaction, as opposed to documenting a combined effect, imposes requirements that most additive studies do not currently meet, and the design choice has to be made before the experiment rather than at analysis. An interaction claim is only interpretable against an explicit null model of non-interaction, and the two candidate nulls answer different questions: concentration addition (Loewe additivity) is appropriate where compounds converge on a common pathway, such as two emulsifiers both thinning mucus, whereas independent action (Bliss independence) is appropriate where mechanisms are distinct, such as an emulsifier and a preservative. The same dataset can appear synergistic against one null and additive against the other, so the null model should be pre-specified and justified mechanistically [79,80]. Design follows from that choice. Factorial designs, in which each compound appears at a single level, support only a test of departure from independent action and have low power for interaction, since the interaction term is estimated from the corner of the design; fixed-ratio (ray) designs, in which a mixture at fixed proportions is tested across a concentration range against the individual concentration-response curves, allow the assumed curve shape to be validated and give substantially stronger interaction claims, and are therefore preferable [79]. Power is the binding constraint at low doses. Interaction terms require considerably larger samples than main effects, and the effect sizes of interest here are small, individually sub-threshold by hypothesis, and measured on readouts with high between-donor and between-animal variance; a study powered for the main effect of each additive will not detect their interaction. Four practical consequences follow. Mixture arms should be powered explicitly for the interaction contrast rather than added to an existing design. The number of compounds should be kept small enough for the interaction to be estimable: realistic co-exposures of five to fifteen additives cannot be resolved combinatorially, so mixtures should be selected by pathway convergence rather than by co-occurrence frequency alone. Composite or multivariate readouts should be pre-registered, to limit multiplicity across many endpoints. And a sham-combination control, in which a compound is combined with itself, should be included where feasible, as a check that the analysis does not generate apparent interaction from the model alone. Where power is insufficient for an interaction test, the honest report is a combined-effect estimate with the interaction left untested, not a claim of additivity.

### 10.4. A Worked Example: Carboxymethylcellulose (E466)

To show the framework is operable rather than aspirational, consider CMC. Tier A: ex vivo human fecal incubation and the Rytter reactor already demonstrate composition and SCFA shifts, with baseline-dependent sensitivity [7,10]—Gate A positive. Tier B: rodent studies show mucus thinning, bacterial encroachment, permeability increase, and low-grade inflammation at dietary-relevant doses, with germ-free reconstitution establishing microbiota-dependence [8,9]—Gate B positive, and specifically flagging that the current “acceptable” status of CMC rests on endpoints that do not include any of these. Tier C: the Chassaing controlled-feeding trial already provides compositional and metabolomic data, including reduced fecal SCFAs and free amino acids, together with mucus encroachment in a subset, but over eleven days, in a sample of sixteen, and without bile-acid quantification [9]; the later five-emulsifier trial adds a four-week placebo-controlled comparison in which CMC lowered SCFA concentrations without changing inflammatory markers [61]. Under the framework, CMC would be a clear candidate for a larger, longer, baseline-stratified Tier C trial with functional co-primary endpoints—a specified next study rather than a general call for “more research”; its cost has not been estimated here. Applied this way, the framework converts the existing CMC literature into a specific regulatory question: does a microbiome and barrier-inclusive evaluation support the current intake status, and what stratified estimates would a re-evaluation need?

### 10.5. Back-Testing the Framework: Three Additives It Has Not Been Built Around

The carboxymethylcellulose example above is open to an obvious objection: CMC has the strongest evidence of any additive considered here, so a framework designed with that evidence in view will pass it. A proof of concept that cannot fail is not a proof of concept. The framework was therefore applied retrospectively to three additives it was not built around, chosen because their evidence profiles differ from CMC’s and from each other: carrageenan (E407), acesulfame K (E950), and titanium dioxide (E171). The three return three different outputs, one of which exposes a structural fault in the protocol as first stated.

Carrageenan (E407) passes both gates, on evidence that is independent of the emulsifier literature. At Tier A, dose-escalating food-grade carrageenan in a staged fecal-microbiota reactor shifts the community toward *Desulfovibrio*, *Bilophila*, and *Ruminococcus* torques and depletes *Bifidobacterium*; critically for the Tier A architecture proposed here, native carrageenan alone does not affect Caco-2 monolayer integrity, whereas supernatants from carrageenan-exposed microbiota cause dose-dependent loss of TEER with reduced ZO-2 and claudin-3/4 [81]. The paired epithelial readout is what detects the effect, and a composition-only screen would have under-called it. At Tier B, dietary κ-carrageenan reduces SCFA and thins the mucus layer without causing overt colitis alone, aggravates infectious colitis, and, in the arm that settles mechanisms, transfers its phenotype by fecal transplant from carrageenan-fed conventional mice but not from carrageenan-fed germ-free mice [82]. Gate B’s microbiota-dependence condition is therefore satisfied directly. Tier C exists but is inadequate: a randomized, double-blind, placebo-controlled trial in ulcerative colitis with twelve completers reported more relapses on carrageenan capsules than on placebo and is too small to be decisive [83]. The framework’s output for carrageenan is thus a specific and non-trivial recommendation, namely an adequately powered, baseline-stratified Tier C trial specifying the molecular-weight fraction tested, rather than a general call for more research.

Acesulfame K (E950) is the equivocal case, and it halts at Gate B. Tier A is positive: dose-escalating acesulfame K in a dynamic simulator seeded with children’s fecal microbiota produces dose-responsive compositional change, a dose-responsive rise in butyrate, and dose-dependent loss of Caco-2 epithelial integrity, attributed to microbial cleavage of acesulfame K to sulfamate and acetoacetate [84]. Two caveats attach to that result. It is currently single-source, and the tested concentrations are not benchmarked to 95th-percentile intake, so it does not in fact satisfy the exposure anchor that Gate A requires. Tier B then fails to confirm. Rodent studies at or near the ADI are null: eight weeks of low-dose exposure up to 15 mg/kg bw/day altered *Clostridium* cluster XIVa and bile-acid metabolism for sucralose but not for acesulfame K [75], and 28 days at 40 and 120 mg/kg bw in rats of both sexes produced only minor fecal microbiome and metabolome changes, with bile-acid shifts read as adaptive [76]. The positive rodent studies either show strongly sex-specific effects or, in the study that tested it, demonstrate intestinal injury and enhanced lymphocyte migration that fecal transfer alone did not reproduce [85], which by the Gate B criterion above identifies a host-mediated rather than a microbiome-mediated effect. No acesulfame-K-specific controlled human trial exists; the best-powered human sweetener trial administered saccharin, sucralose, aspartame, and stevia, not acesulfame K [11].

The framework therefore returns “Tier A positive, Gate B not satisfied”: hold, with a documented data gap, and no advance to Tier C. Set against EFSA’s 2025 re-evaluation, which established an ADI of 15 mg/kg bw/day in place of the earlier 9 mg/kg bw/day and found exposure generally below the ADI in all population groups [46], that is a defensible output. It is also worth stating plainly that Gate A here arguably over-called on a single unanchored study, which is the opposite failure mode from the one that follows.

Titanium dioxide (E171) is the case where the protocol gets it wrong, and the reason is structural rather than statistical. At Tier A, exposure of a defined human intestinal community to two food-grade E171 formulations at 100–250 ppm, explicitly set at the intestinal concentration expected after eating one or two pieces of gum or candy, produced no effect on gas production, only minor fatty-acid shifts, and a single modest taxon change [86]. On that evidence Gate A as originally formulated exits E171 with a documented negative at realistic exposure. The exit is wrong. In vivo, food-grade TiO_2_ is found inside Peyer’s-patch immune cells, decreases regulatory T cells, skews splenic responses toward Th1 and Th17, and after 100 days produces colonic micro-inflammation and preneoplastic lesions at human-relevant doses [87]; in a separate model it leaves small-intestinal and colonic composition largely unchanged while reducing Muc2, shortening crypts, and recruiting CD8^+^ T cells and macrophages [88].

Two conclusions follow. A cell-free fermentation vessel cannot express a mechanism that runs through particle uptake into gut-associated lymphoid tissue, so for particulates the Tier A negative is uninformative by construction rather than reassuring; and compositional nullity is not a valid surrogate even in vivo, since E171 can be close to silent on composition while damaging the host. This is the justification for the categorical exemption added to Gate A in Section 10.1, routing particulates and nanomaterials directly to Tier B. It also sets a limit on the Gate A exit in general: adding a host-tissue co-culture arm to Tier A narrows the gap but does not close it, because epithelial co-cultures still contain no immune compartment.

Taken together the back-test does what the CMC example cannot. Three additives yield three outputs: proceed to a specified Tier C trial (carrageenan), hold at Gate B with a documented gap (acesulfame K), and bypass Gate A entirely as a particulate (titanium dioxide). The protocol therefore discriminates between compounds rather than confirming a prior. It also shows that its errors are not symmetrical: soluble chemicals risk over-calling at Gate A on thin Tier A evidence, particulates risk under-calling, and the correction for each is different. One further limitation emerged from the exercise and is conceded here. No existing Tier A study of any of these three compounds reports concentrations benchmarked to 95th-percentile population intake across a defined donor panel, which is what Gate A as specified requires. That benchmark, not the sequencing or the barrier assay, is currently the binding obstacle to operationalizing Tier A.

### 10.6. Reformulation and the Microbiome Profile of Replacement Ingredients

A framework that can identify microbiome-active additives raises the question of what replaces them, and reformulation is where any regulatory tightening would be felt. The replacement literature is small but no longer absent. Reformulated cooked turkey breast and ham in which additives and allergens were replaced by natural extracts, vinegar, and inulin improved protein digestibility and DIAAS protein quality and increased total SCFA production in in vitro colonic fermentation with fecal inocula from normal-weight and obese donors, while leaving phylum- and genus-level composition largely unchanged [89]; in a subsequent five-week double-blind randomized trial of the same products, oxidative and inflammatory markers differed between arms for TNF-α and IL-1β, and the intervention group showed reduced nitrate excretion and a decrease in nitrification-related gut bacteria [90]. This is close to the endpoint set proposed above, applied to a substitution rather than to an additive, and it illustrates that the same assays serve both directions of the question.

Two cautions should be attached. First, substitution is not inherently benign. Natural additives are not safer than synthetic ones by virtue of origin, and the same microbiome and barrier endpoints are warranted before a replacement is treated as an improvement [91]. Plant extracts and essential oils used as antimicrobial replacements have, by design, antimicrobial activity that does not stop at the target organism, and clean-label nitrite alternatives have, in at least one in vitro colonic-fermentation study, produced no detectable benefit over conventional formulations on SCFA or community composition. Second, the evaluation route for a replacement is the same tiered one. A recent application of a standardized in vitro gastrointestinal and colonic-fermentation model to candidate texturizers illustrates the point: chitosan preparations promoted *Clostridium* cluster IV and *Bifidobacteriaceae* and increased butyrate and propionate, whereas hydroxypropyl methylcellulose reduced *Lactobacillaceae* and raised undesirable metabolites, so a substitution that looks equivalent technologically separates clearly on microbiome endpoints [92]. Reformulation therefore belongs inside the framework rather than beside it: a replacement should enter Tier A on the same terms as the compound it displaces, and a replacement that has not been screened should not be presented as a microbiome-sparing choice.

### 10.7. Feasibility

Adding microbiome endpoints to regulatory studies raises real challenges: sequencing and metabolomic pipelines are not yet standardized for regulatory use, inter-laboratory variability is substantial, and decision thresholds for “adverse” microbiome change are not established. These are the very problems the EFSA roadmap [22] and microorganism guidance [23] are beginning to address, and the tiered design above is intended to resolve additives at the lowest tier that can answer the question, with the exemption for particulates noted in Section 10.1. Whether that reduces aggregate cost is an empirical claim that this review does not substantiate, and the proportion of additives that a Tier A screen would in fact resolve is unknown: the back-test in Section 10.5 found that none of the three additives examined had Tier A data anchored to 95th-percentile intake, so the gate has not yet been applied prospectively to any compound. A formal cost analysis, and ring-trials to establish endpoint reproducibility and decision thresholds, are prerequisites before any element of this framework could be presented to regulators as ready for adoption. Because the effect sizes that motivate the framework are small, a proportionality assessment that weighs the cost of universal Tier A screening against the absolute risk it would avert belongs among these prerequisites. It is presented as an implementable research-and-policy agenda with those prerequisites made explicit, not as a finished instrument.

## 11. Conclusions

This review brings together mechanistic, translational, and epidemiological evidence for treating commonly used food additives as context-dependent, microbiome-active exposures rather than uniformly inert ingredients. The central argument is that classical toxicological assessment, built on direct cytotoxicity, genotoxicity, carcinogenicity, and reproductive endpoints, is not designed to detect the microbiome-mediated effects that additives can produce at dietary-relevant doses, and that the tools to detect them now exist.

The strongest evidentiary case concerns gastrointestinal and metabolic outcomes. For gastrointestinal disease the support is genuinely multi-stranded, combining mechanistic causal models, case–control exposure data, and dietary-exclusion evidence. Controlled human trials demonstrate microbiome-dependent effects on surrogate endpoints, not on incident disease. Autoimmune, neurological, and psychiatric associations remain preliminary and largely animal derived.

The framework translates this into an operational contribution: a tiered, decision-gated protocol for microbiome-inclusive additive evaluation, with a defined minimum endpoint set, exposure-realistic and mixture-aware design requirements, a susceptibility-stratification component, and a single additive worked through it as proof of concept. This extends, rather than restates, the direction already set by EFSA’s microbiome roadmap by making it additive-specific and operable. The evidence supports considering microbiome-relevant endpoints in additive safety evaluation and sets out one way of specifying them; given a narrative review with single screening and non-blinded grading, a stronger verb would not be warranted by the methodology, whatever the mechanistic case; it does not yet establish additive-specific causation for most chronic-disease outcomes, and the priority now is the exposure-realistic, baseline-stratified, functionally instrumented human and mixture studies that would close that gap.

Four points carry the argument. First, the biology is real but narrower than the literature implies: emulsifiers and non-nutritive sweeteners have direct human and ex vivo evidence of microbiome and barrier effects at doses people plausibly consume, and the effects are individual rather than uniform. Second, the epidemiology is thinner than the mechanism: the prospective additive-specific signal for metabolic and cardiovascular outcomes comes from one cohort, with indirectly estimated exposure and hazard ratios near unity, and null human trials exist for both emulsifiers and sweeteners. Third, what follows for assessment is a question of endpoints rather than of bans: composition alone is uninformative, and composition together with a functional output and a barrier or immune readout is what distinguishes an adaptive microbiome response from an adverse one. Fourth, the proposed protocol discriminates between compounds, as the back-test shows, but it fails for particulates unless they bypass its first gate, and no additive yet has Tier A data anchored to population intake as the protocol requires. Causal attribution to incident chronic disease remains unestablished for every domain considered here.

## Figures and Tables

**Table 1:** What this review adds, compared to prior syntheses and current regulatory activity.

Prior Work	What It Provides	What This Review Adds
Whelan et al. (2024) [6] (Nat Rev Gastroenterol Hepatol)	Comprehensive catalog of additive effects on gut health, organized by additive class	Pathway-based organization; separate grading of association/plausibility/causation; effect sizes with CIs; integration of mixture epidemiology and null trials
EFSA GI-microbiome roadmap [22] (2024)	General, cross-sector roadmap for integrating gut microbiomes into risk assessment	An additive-specific, decision-gated protocol with explicit pass/fail gates and a worked case study (CMC)
EFSA microorganism guidance [23]; toxicomicrobiomics [24]	Principle that microbiome endpoints belong in food-safety evaluation; characterization methods	A defined minimum endpoint set mapped to each mechanistic pathway (Section 10.2), tiered by evidence requirement
Cox et al. (2021) [25]	Notes microbiome endpoints absent from emulsifier evaluation	Extends beyond emulsifiers to six classes; specifies which endpoints and at which tier
Prior additive reviews [16,17,18,19,20,21]	Class-level inventories of effects	Explicit hazard/exposure/risk separation; dose–ADI-intake comparison (Section 9); susceptibility axis bounded to classes with direct evidence

**Table 3:** Classification linking additive class to primary microbiome-relevant interaction mode and highest evidence tier.

Additive Class	Key Compounds (E-Number)	Primary Interaction Mode	Highest Evidence Tier
Non-nutritive sweeteners	Saccharin, sucralose, aspartame, acesulfame K, stevia (E950-E969)	Functional modulation: community restructuring, SCFA/metabolite change; inter-individual response heterogeneity	Controlled human RCT [11]
Emulsifiers	CMC (E466), P80 (E433), lecithin (E322), sucrose esters, mono-/diglycerides (E471), carrageenan (E407)	Mucus–epithelial impairment; barrier disruption; bacterial encroachment	Controlled human feeding trial [9]
Preservatives	Sodium benzoate (E211), potassium sorbate (E202), nitrite (E250)	Direct antimicrobial pressure; fermentation loss; compound-specific restructuring	In vivo animal, multi-compound [39]
Colorants/particulate	Allura Red AC (E129), tartrazine (E102), TiO_2_ (E171)	Serotonin-mediated immune sensitization (dyes); spatial-functional disruption (particles)	In vivo animal + causal transfer [40]
Thickeners/stabilizers	Xanthan gum (E415), carrageenan (E407), maltodextrin	Selective-substrate ecology; mucin depletion; ER-stress barrier vulnerability	In vivo animal; ex vivo human [41,42]
Flavor enhancers	Monosodium glutamate (E621)	Context-dependent ecological restructuring; gut–brain modulation via glutamate-GABA	Ex vivo human fecal co-culture [43]

**Table 4:** Food additives and intestinal-barrier disruption, by mechanism and study model.

Barrier Mechanism	Additive (Class)	Study Model (Tier)	Key Finding	Ref.
Mucus thinning/bacterial encroachment	CMC, P80 (emulsifiers)	Mouse in vivo; ex vivo human fecal (T2–3)	Reduced mucus thickness; bacteria closer to epithelium	[7,9]
Synergistic mucus/barrier impairment	Carrageenan + high sugar	Mouse in vivo + in vitro (T1,3)	Synergistic MUC2 reduction; *Akkermansia* depletion	[58]
Goblet-cell ER stress/mucin depletion	Maltodextrin (bulking agent)	Mouse in vivo (T3)	Impaired MUC2 secretion; accelerated colitis	[13]
Tight-junction disruption (receptor-mediated)	Saccharin, sucralose, aspartame (NNS)	Caco-2 monolayer (T1)	Reduced TEER via T1R3; loss of claudin/occludin (Tier 1; colonic delivery expected for sucralose only, not for acesulfame K, aspartame or saccharin)	[59]
Increased paracellular permeability	P80 (emulsifier)	Mouse in vivo (T3)	Leaky gut; skeletal-muscle glucose impairment	[60]

**Table 5:** Graded evidence for additive-associated chronic-disease risk across six domains. Grades applied per the pre-specified anchors by two raters (raw agreement 15/18 cells; sensitivity analysis in text). Labels describe evidentiary maturity, not a recommendation for action.

Disease Domain	Key Implicated Classes	Association	Mechanistic Plausibility	Causal Confidence	Evidentiary Maturity ^4^
Gastrointestinal (IBD, CRC, IBS) ^3^	Emulsifiers, carrageenan, preservatives	Moderate ^2^	Strong	Moderate	Most mature
Metabolic (T2D, obesity)	Emulsifiers, NNS	Moderate ^1^	Strong	Moderate	Intermediate maturing
Cardiovascular (CVD, CHD)	Emulsifiers, NNS, nitrites	Moderate	Moderate	Low	Intermediate
Autoimmune (RA, SLE, MS)	Emulsifiers, colorants, NNS	Weak	Moderate	Low	Preliminary
Neurological (cognitive decline, Parkinson’s)	Emulsifiers, NNS (indirect)	Weak	Emerging	Low	Preliminary
Psychiatric (depression, anxiety, ADHD)	Aspartame, colorants, MSG	Weak	Emerging	Low	Preliminary

^1^ Down-graded from a naïve “Strong” reading because the prospective signal, though internally consistent, derives from a single cohort (NutriNet-Santé) and therefore does not meet the ≥2-independent-cohort anchor; effect sizes are modest (per-increment HR ≈ 1.03–1.15) and exposure is estimated indirectly. ^2^ The pre-specified anchor for a Strong association requires consistent, replicated associations in at least two independent large prospective cohorts or a meta-analysis. The gastrointestinal evidence does not meet that anchor: it rests on Tier 3 causal animal models, a case–control exposure comparison [37], and a scoping review of dietary-exclusion studies [38], none of which is a prospective cohort of additive intake with incident disease. Breadth of evidence across tiers is not strength of association as defined here, and the domain is graded Moderate accordingly. Mechanistic plausibility remains Strong and causal confidence Moderate, and the domain retains the highest evidentiary maturity because maturity reflects cross-tier convergence rather than associational strength alone. ^3^ The domain aggregates three conditions with materially different evidence. For IBD there are converging Tier 3 causal models, case–control exposure data, and dietary-exclusion evidence; taken alone, IBD would be graded association Moderate, plausibility Strong, causal confidence Moderate. For colorectal cancer the evidence is mechanistic and indirect, with no additive-specific prospective cohort: association Weak, plausibility Moderate, causal confidence Low. For IBS the additive-specific evidence is the sparsest of the three: association Weak, plausibility Emerging, causal confidence Low. The single domain-level row is retained for comparability with the other five domains, but it is driven by IBD and should not be read as applying equally to CRC or IBS. ^4^ Rule linking the three dimensions to evidentiary maturity, applied uniformly across domains: “Most mature” requires plausibility Strong and causal confidence at least Moderate, with at least one Tier 5 human intervention study on a relevant endpoint; “Intermediate” requires plausibility at least Moderate and association at least Moderate; “Preliminary” applies where causal confidence is Low and the association rests on animal or indirect human evidence. The sensitivity analysis moved each of the eighteen cells by one adjacent level in turn; no single move reassigns any domain between these three tiers.

**Table 6:** Direction-of-effect (SWiM-style) summary across the six disease domains. This is a qualitative synthesis of the direction and consistency of the reported effects, not a quantitative pooling; it complements the graded matrix (Table 5) and follows the direction-of-effect approach adopted here [31].

Disease Domain	Contributing Evidence (Tier)	Direction of Effect	Consistency of Direction	Discordant or Null Evidence
Gastrointestinal (IBD, CRC)	Tier 3 causal animal models; case–control [37]; dietary-exclusion [38]; mechanistic [6]	Toward harm	Consistent across independent strands	Case–control open to reverse causation; no incident-disease RCT
Metabolic (T2D, obesity)	One prospective cohort [36,70,71]; glycemic-surrogate RCT [11]	Toward harm	Internally consistent within a single cohort	Null within-ADI microbiome RCT [12]; not replicated in an independent cohort
Cardiovascular (CVD)	One prospective cohort [64,65,72]; immune-metabolic mechanism	Toward harm	Single cohort; no independent replication	No RCT; residual dietary-pattern confounding
Autoimmune (RA, SLE, MS)	Immune-mechanistic reasoning [6,40]; emerging UPF-cohort signals	Toward harm (sparse)	Weak; few direct data	No interventional evidence; additive-specific attribution absent
Neurological (cognitive decline, Parkinson’s)	Animal models [66]; indirect human synthesis [6]	Toward harm (animal)	Animal-only; dose-uncertain	Human additive-specific data lacking
Psychiatric (depression, anxiety, ADHD)	Animal models [40,43,66]	Toward harm (animal)	Animal-only; dose-uncertain	Human disease-endpoint data lacking

**Table 7:** Tested doses in principal studies versus acceptable daily intake (ADI) and estimated human exposure. ADI values were re-checked against the primary FDA, EFSA, and JECFA evaluations; “-” marks values that are not established or cannot be reliably determined from the source. In the dose-realism column, “realistic” means only that the dose administered in the source study falls within or below the ADI, or was described by its authors as dietary-anchored. It is not a verified comparison against measured population intake, which is unavailable for most of these compounds; cells with “-” in the intake column are those for which no quantitative human-intake estimate was located, and for those compounds no claim about exposure realism can be substantiated beyond the ADI comparison.

Compound (E-Number)	Principal Study & Dose	ADI (Source)	Estimated Human Intake	Dose-Realism Verdict
CMC (E466)	Chassaing [9]: ~15 g/day, 11 days (human trial); ≈214 mg/kg bw/day for a 70-kg adult	“Not specified”/acceptable, no numeric ADI	Not systematically quantified for high consumers	High consumer to supraphysiological. The dose was administered to humans, but was selected to model high-end rather than typical exposure; EFSA set no numerical ADI for celluloses, and no systematic population intake estimate for E466 is available against which to judge it
Polysorbate-80 (E433)	Panyod [8], Nishimura [60]: ~1% diet (mouse)	Group ADI for polysorbates ~25 mg/kg bw/day (EFSA)	-	Dose within the group ADI; no population intake estimate available for comparison
Carrageenan (E407)	Gao [58], Bellanco [42]: dietary-anchored (mouse)	Temporary ADI 75 mg/kg bw/day (EFSA 2018), low-MW fraction flagged [47,48]	-	Realistic; specify MW fraction
Sucralose	Suez [11]: 1–3 servings/day (human); Chi [55] chronic (mouse)	5 (FDA)/15 (EFSA) mg/kg bw/day	Typically, below ADI	Realistic (human); mouse chronic variable
Aspartame	Jones [66]: 8–15% of FDA max (mouse); Suez [11] servings (human)	50 (FDA)/40 (EFSA, JECFA) mg/kg bw/day [45]	Below ADI in most surveys	Dose realistic (human trial); neurobehavioral findings based on a single rodent model
Acesulfame K	Rodent GBA studies [6]; low-dose rodent studies at or below the ADI [75,76]	15 mg/kg bw/day (FDA); 15 mg/kg bw/day (EFSA 2025, replacing the former SCF value of 9 mg/kg bw/day) [46]	Below ADI	Variable
Allura Red AC (E129)	Kwon [40]: “dietary-relevant” (mouse)	7 mg/kg bw/day (EFSA)	-	Reported as dietary-relevant by the source study; no independent intake estimate available for comparison
Titanium dioxide (E171)	Multiple (mouse) [51]	No ADI; EFSA 2021 “no longer safe”; EU ban 2022 [52]	Frequently below tested doses	Often supra-physiological
Sodium benzoate (E211)	Li [39], Nagpal [49]: dietary range (mouse); de Souza Lopes [44] in vitro	5 mg/kg bw/day (benzoic acid)	-	Mixed; some in vitro supra-physiological
Maltodextrin	Zangara [13]: dietary-anchored (mouse)	No numeric ADI (GRAS/acceptable)	-	Realistic to moderate
MSG (E621)	Rodent, heterogeneous [53]	Group ADI 30 mg/kg bw/day (as glutamic acid)	Often near/above at high intake	Frequently above typical intake

**Table 8:** Mechanistic pathway mapping and microbiome-inclusive endpoints recommended for each.

Biological Pathway	Converging Additive Classes	Key Mechanistic Evidence	Downstream Host Outcome	Recommended Regulatory Endpoint(s)
Microbial community restructuring	Emulsifiers, NNS, preservatives, colorants	Ex vivo fecal screening [7,15]; in vivo multi-compound [8,39,49]	Altered SCFA/metabolite profiles; reduced colonization resistance	16S/shotgun composition (α/β-diversity, key taxa)
SCFA/fermentative capacity	Emulsifiers, NNS, preservatives, thickeners	Functional fermentation assays [15]; mouse models [8,55]	Epithelial fuel deficit; impaired TJ signaling; insulin resistance	Targeted fecal SCFAs (GC/NMR)
Bile-acid biotransformation	NNS (sucralose), emulsifiers	Sucralose-FXR mechanism [55]; emulsifier bile-acid shifts [8]	Lipid/cholesterol dysregulation; hepatic disturbance	Bile-acid pool profiling (primary/secondary)
Tryptophan-indole-serotonin	Colorants (Allura Red), emulsifiers (P80)	Serotonin-colitis causation [40]; AhR/ILC3 impairment [6]	Intestinal inflammation; immune dysregulation; gut–brain effects	Indole/AhR-ligand metabolites; mucosal IL-22
Mucus-barrier integrity	Emulsifiers, carrageenan, maltodextrin, NNS	Mucus thinning [7,9]; ER stress [13]; T1R3 permeability [59]; co-exposure synergy [58]	Endotoxemia; systemic low-grade inflammation	In vivo permeability (FITC-dextran); TJ histology; FABP2/LBP

## Data Availability

No new data were created or analyzed in this study. All data discussed are available in the cited published sources.

## Supplementary Materials

The following supporting information can be downloaded at: https://www.mdpi.com/article/10.3390/nu18213461/s1; Table S1: Search domains, representative terms, and selection criteria; Text S1: Full database search strategies; Table S2: ROBINS-E risk-of-bias appraisal of the pivotal NutriNet-Santé analyses; Text S2: ROBINS-E appraisal: domain-level justifications; Text S3: Rationale for the Tier A donor panel and the Gate A thresholds; Text S4: Inventory of included study types.

## Abbreviations

The following abbreviations are used in this manuscript:

ADHD	attention-deficit/hyperactivity disorder
ADI	acceptable daily intake
AhR	aryl hydrocarbon receptor
CI	confidence interval
CMC	carboxymethylcellulose (E466)
CRC	colorectal cancer
CVD	cardiovascular disease
EFSA	European Food Safety Authority
ER	endoplasmic reticulum
FABP2	fatty acid-binding protein 2
FDA	U.S. Food and Drug Administration
FITC-dextran	fluorescein isothiocyanate–dextran
FXR	farnesoid X receptor
GABA	γ-aminobutyric acid
GBA	gut–brain axis
GI	gastrointestinal
GRADE	Grading of Recommendations, Assessment, Development and Evaluations
GRAS	generally recognized as safe
HPA	hypothalamic–pituitary–adrenal (axis)
HR	hazard ratio
IBD	inflammatory bowel disease
IL	interleukin (e.g., IL-22)
ILC3	group 3 innate lymphoid cell
JECFA	Joint FAO/WHO Expert Committee on Food Additives
LBP	lipopolysaccharide-binding protein
LPS	lipopolysaccharide
MeSH	Medical Subject Headings
MSG	monosodium glutamate (E621)
MUC2	mucin 2
NF-κB	nuclear factor kappa B
NLRP3	NOD-, LRR- and pyrin domain-containing protein 3
NMR	nuclear magnetic resonance
NNS	non-nutritive sweetener(s)
NOVA	NOVA food-processing classification (name, not an acronym)
P80	polysorbate 80 (E433)
PRESS	Peer Review of Electronic Search Strategies
PRISMA	Preferred Reporting Items for Systematic Reviews and Meta-Analyses
PRISMA-S	PRISMA literature Search extension
RCT	randomized controlled trial
RoB	risk of bias
ROBINS-E	Risk Of Bias In Non-randomized Studies—of Exposures
SANRA	Scale for the Assessment of Narrative Review Articles
SCFA	short-chain fatty acid
SHIME	Simulator of the Human Intestinal Microbial Ecosystem
SWiM	Synthesis Without Meta-analysis
T1R3	taste receptor type 1 member 3 (sweet-taste receptor)
T2D	type 2 diabetes
TEER	transepithelial electrical resistance
TiO_2_	titanium dioxide (E171)
TJ	tight junction
TLR4	Toll-like receptor 4
TNF	tumor necrosis factor
UPF	ultra-processed food
WHO	World Health Organization
ZO-1	zonula occludens-1
