# Supplementary material for: Food Additives as Microbiome-Active Dietary Exposures: Mechanisms, Host Susceptibility, and an Operational Case for Microbiome-Inclusive Risk Assessment
Source: Nutrients. 2026 Oct 22;18(21):3461. doi: 10.3390/nu18213461 (PMC13764718; doi:10.3390/nu18213461)
Supplement: Supplementary file 1 [file nutrients-18-03461-s001.zip › nutrients-4612821-supplementary.pdf]

nutrients

MDPI

Supplementary Materials

Table S1. Search domains, representative terms, and selection criteria

Element	Content
Databases	PubMed/MEDLINE; Embase (Ovid); Scopus; Web of Science Core Collection
Date window	Jan 2020 - Jun 2026 (earlier only if foundational and without a recent equivalent)
Domain I - Exposure	"food additives", "dietary emulsifiers", "non-nutritive/artificial sweeteners", CMC, polysorbate-80, carrageenan, maltodextrin, titanium dioxide, sodium benzoate, sucralose, aspartame, "ultra-processed food"
Domain II - Microbiota	"gut/intestinal microbiome", "microbial diversity", "dysbiosis", "short-chain fatty acids", "bile acid metabolism", "tryptophan metabolism"
Domain III - Host mechanism	"intestinal barrier", "tight junction", "mucus layer", "innate immunity", "inflammasome", "NF-κB", "gut-brain axis"
Domain IV - Outcome	"inflammatory bowel disease", "metabolic syndrome", "type 2 diabetes", "cardiovascular disease", "colorectal cancer", "obesity", "autoimmune/neurological disorders"
Inclusion	Original animal/in vitro/ex vivo/human studies reporting microbiome endpoints; high-quality reviews; regulatory/methodological sources on microbiome endpoints
Exclusion	No microbiome endpoint or endpoint not attributable to additives; macronutrient/caloric focus only; abstracts/editorials/letters without primary data; supra-physiological dosing without human-relevance discussion; non-English
Reporting standard	SANRA (narrative review); no PRISMA completeness claim

Text S1. Full database search strategies

Reported per the PRISMA-S search-reporting extension. The strategies below are those executed on each platform; Section 2 of the main article describes how the concept blocks were combined.

Documentation table. The strategy was built on the term set in Table S1. The primary combination was Block A (exposure) AND Block B (microbiota); a focused variant added Block C (host mechanism / outcome). Consistent with the recommendation to avoid more than three concept blocks, the population block was not used as a separate restrictor. As a narrative integrative review, exhaustive per-database record counts were not tabulated; the strategies are provided so the search is reproducible.

Field	Content
Databases	MEDLINE (PubMed); Embase (Ovid); Scopus; Web of Science Core Collection
Coverage	database inception to Jun 2026 (primary window Jan 2020 - Jun 2026)
Date searched	June 2026
Language	English (translation obtained at full text where needed)
Filters	none applied at the search stage. Animal studies were eligible and form a substantial part of the evidence synthesised here; no animal-exclusion filter was applied. A validated animal search filter was used only to tag records by evidence tier during screening, not to remove them.
Supplementary sources	citation chaining from key reviews; EFSA Journal, JECFA monographs, and FDA GRAS notices hand-searched. Grey literature, conference abstracts, and trial registries were not searched systematically
Records retrieved	not exhaustively tabulated (narrative review)

A. MEDLINE (PubMed) - primary strategy.

#A "Food Additives"[Mesh] OR "food additive*"[tiab] OR "dietary emulsifier*"[tiab]
OR emulsifier*[tiab] OR "non-nutritive sweetener*"[tiab] OR "artificial sweetener*"[tiab]
OR "non-caloric sweetener*"[tiab] OR carboxymethylcellulose[tiab]
OR "carboxymethyl cellulose"[tiab] OR E466[tiab] OR polysorbate*[tiab] OR P80[tiab]
OR carrageenan*[tiab] OR maltodextrin[tiab] OR "titanium dioxide"[tiab] OR E171[tiab]
OR "sodium benzoate"[tiab] OR sucralose[tiab] OR aspartame[tiab] OR acesulfame*[tiab]
OR saccharin[tiab] OR "monosodium glutamate"[tiab] OR "xanthan gum"[tiab]
OR "guar gum"[tiab] OR "ultra-processed food*"[tiab] OR "ultraprocessed food*"[tiab]

#B "Gastrointestinal Microbiome"[Mesh] OR "gut microbiota"[tiab] OR "gut microbiome"[tiab]
OR "intestinal microbiota"[tiab] OR "intestinal microbiome"[tiab] OR dysbiosis[tiab]
OR "microbial diversity"[tiab] OR "Fatty Acids, Volatile"[Mesh]
OR "short-chain fatty acid*"[tiab] OR SCFA[tiab] OR "bile acid*"[tiab]
OR "tryptophan metabol*"[tiab] OR indole*[tiab]

#C "intestinal barrier"[tiab] OR "tight junction*"[tiab] OR "mucus layer"[tiab]
OR permeability[tiab] OR inflammasome[tiab] OR "NF-kappa B"[tiab]
OR "gut-brain axis"[tiab] OR "Inflammatory Bowel Diseases"[Mesh]
OR "inflammatory bowel disease*"[tiab] OR "type 2 diabetes"[tiab]
OR "cardiovascular disease*"[tiab] OR "colorectal cancer"[tiab] OR obesity[tiab] OR "metabolic syndrome"[tiab]

#D #A AND #B (primary retrieval)

#E #A AND #B AND #C (focused retrieval)

B**. Embase (Ovid) - translated (adj proximity; .ti,ab,kw. fields).**

1 'food additive'/exp OR (emulsifier* OR 'non-nutritive sweetener*' OR carrageenan*
OR polysorbate* OR carboxymethylcellulose OR maltodextrin OR 'titanium dioxide'
OR sucralose OR aspartame OR acesulfame* OR 'ultra-processed food*').ti,ab,kw.

2 'intestinal flora'/exp OR (microbiota OR microbiome OR dysbiosis
OR 'short chain fatty acid*' OR 'bile acid*' OR tryptophan).ti,ab,kw.

3 1 AND 2

4 limit 3 to (human or animal) and yr="1980 -Current"

C. Scopus.

TITLE-ABS-KEY(("food additive*" OR emulsifier* OR "non-nutritive sweetener*" OR carrageenan* OR sucralose OR aspartame OR "ultra-processed food*") AND (microbiota OR microbiome OR dysbiosis OR "short-chain fatty acid*" OR "bile acid*"))

D. Web of Science Core Collection.

TS=(("food additive*" OR emulsifier* OR "non-nutritive sweetener*" OR carrageenan* OR sucralose OR aspartame OR "ultra-processed food*") AND (microbiota OR microbiome OR dysbiosis OR "short-chain fatty acid*" OR "bile acid*"))

Note: truncation (e.g. emulsifier*, sweeten*) was checked for false hits before inclusion; in PubMed both a controlled-vocabulary (MeSH) layer and a free-text (tiab) layer were used, because truncation disables automatic MeSH mapping. The strategy was checked against a reference set of key studies; entries that were not retrieved were used to add missing terms. Independent PRESS-style peer review of the search is recommended before any future systematic-review use.

Table S2. ROBINS-E risk-of-bias appraisal of the pivotal NutriNet-Santé analyses The prospective additive-specific evidence for metabolic and cardiovascular outcomes rests on the NutriNet-Santé cohort. These analyses were appraised with ROBINS-E across its seven domains. The overall judgement follows the least favorable domain. Judgements are the appraisers' own and are based on the published reports. The risk-of-bias profiles are near-identical across these analyses, which follows from their design: the four analyses draw on the same cohort, the same repeated 24-h dietary records, and the same additive-composition matching procedure, so the dominant limitation (D2, measurement of exposure) is shared by construction.

Where the analyses differ, they differ in outcome ascertainment rather than in exposure, and D6 is rated Low for all. Debras 2022 and Debras 2023 [65,71] are appraised as one row because they apply a single exposure-assessment protocol in the same cohort to two outcome sets; their study-specific judgements are nonetheless identical on every domain, and separating the rows would duplicate rather than refine the appraisal.

Study	D1	D2	D3	D4	D5	D6	D7	Overall
Salame 2024 (emulsifiers → T2D) [70]	Some concerns	High	Some concerns	Some concerns	Some concerns	Low	Low	High
Sellem 2023 (emulsifiers → CVD) [64]	Some concerns	High	Some concerns	Some concerns	Some concerns	Low	Low	High
Debras 2022/2023 (sweeteners → CVD/T2D) [65,71]	Some concerns	High	Some concerns	Some concerns	Some concerns	Low	Low	High
Payen de la Garanderie 2025 (mixtures → T2D) [36]	Some concerns	High	Some concerns	Some concerns	Some concerns	Low	Low	High

D1 confounding; D2 measurement of exposure; D3 selection of participants; D4 post-exposure classification; D5 missing data; D6 measurement of outcome; D7 selective reporting.

Text S2. ROBINS-E appraisal: domain-level justifications

Domain justifications (common to the NutriNet-Santé analyses).

D1 Confounding - Some concerns. Analyses adjust extensively for age, sex, energy intake, dietary quality, physical activity, smoking, and socioeconomic indicators, but residual confounding by the overall ultra-processed dietary pattern and by unmeasured lifestyle factors remains plausible, and additives are strongly correlated with one another and with that pattern. Because additive intake is largely a marker of the overall ultra-processed dietary pattern, statistical adjustment cannot fully separate an additive-specific effect from the pattern it indexes, so this domain is judged conservatively.

D2 Measurement of exposure - High. Additive intake is not measured; it is estimated by matching repeated 24-h dietary records to food-composition and commercial brand databases. Product reformulation over time, brand-level heterogeneity, and the absence of any biomarker introduces substantial, largely non-differential misclassification. This is the domain that drives the overall rating.

D3 Selection of participants - Some concerns. The cohort is a self-selected online volunteer sample, predominantly women and more highly educated and health-conscious than the general population, which limits generalizability; incident-design entry mitigates but does not remove selection effects.

D4 Post-exposure classification - Some concerns. Diet is time-varying; exposure is frequently summarized over an early window, so changes during follow-up may be imperfectly captured.

D5 Missing data - Some concerns. Loss to follow-up and incomplete records are handled by the authors, but the potential for informative missingness is not fully excluded.

D6 Measurement of outcome - Low. Type 2 diabetes is ascertained through multiple linked sources including medication reimbursement; cardiovascular events are validated by an expert adjudication committee.
D7 Selective reporting - Low. Analyses are pre-specified and consistently reported, with multiple sensitivity analyses.
Implication. A high overall risk of bias, driven by exposure measurement and compounded by the near collinearity of additive intake with the overall ultra-processed dietary pattern (D1), is the principal reason the GRADE certainty for the metabolic and cardiovascular outcomes does not exceed low (Table 2). It also supports the review's central methodological argument: population-average estimates from indirect exposure assessment under-specify the true additive-specific risk, and biomarker-anchored, exposure-realistic studies are the priority.

Text S3. Rationale for the Tier A donor panel and the Gate A thresholds

Donor panel. Donors are run individually rather than pooled because pooling raises apparent richness and reduces between-sample variance. That is desirable when the object is a reproducible average response, but it defeats the purpose of Tier A, which is to detect whether any recognizable human microbiota configuration responds to the compound. A panel of twelve is proposed as the smallest number that allows three donors per dominant enterotype with two low-richness profiles included, and that supports the "at least three of twelve donors" rule used at Gate A.

It is a pragmatic minimum rather than a formally powered figure, and a panel sized from pilot variance estimates for the specific assay would be preferable where those estimates exist. Enterotype stratification is used instead of random sampling because the compositional space is strongly clustered and random recruitment of twelve donors from a single population will usually over-represent the locally dominant configuration. Low-richness, low-cell-count profiles of the *Bacteroides*-2 type are included deliberately: in faecal-transfer trials these are the profiles associated with weaker colonization resistance and with discordant treatment responses, and a panel that excludes them will systematically under-detect susceptibility-dependent effects. Characterization for bile-salt-hydrolase carriage, butyrate-synthesis capacity and mucin-degrading taxa is specified because these are the functional guilds through which the mechanisms identified in Sections 4 and 5 of the main article operate. Guild composition also predicts the direction of the bile-acid and short-chain-fatty-acid readouts more reliably than taxonomic assignment does.

Gate A thresholds. The three thresholds are set so that each is measurable against a vehicle-matched control within a single run, and so that none of them depends on a nominal significance test whose power varies with the number of donors screened. The compositional criterion is expressed against the assay's own donor-specific technical variance rather than as a fixed distance. Within-donor technical replication in these systems typically resolves differences far smaller than those that are biologically meaningful, so a nominal p-value will flag effects of no consequence. The 20% reduction in total or butyrate short-chain fatty acid concentration and the 15% fall in transepithelial electrical resistance are indicative values chosen to sit above typical inter-run variation in these assays while remaining below the effect sizes reported for the emulsifiers with the strongest evidence. They are offered as a starting point for ring-trial calibration, not as validated decision limits, and establishing them empirically is one of the prerequisites set out in Section 10.7 of the main article. The requirement for reproduction in an independent run addresses the principal failure mode of single-run screening, which is that a donor-specific perturbation in one reactor is indistinguishable from a compound effect.

Why the Gate A exit is restricted. A Tier A negative is evidence that a compound does not act on the microbiota or on microbiota-proximal barrier function at the concentrations tested. It is not evidence of safety, and three mechanisms escape it entirely: effects requiring host cells absent from the system, effects requiring an intact immune compartment, and effects of metabolites generated only after absorption. The back-test in Section 10.5 of the main article shows the second of these producing a false negative for titanium dioxide, which is the basis for the categorical exemption of particulates and nanomaterials in Section 10.1.

Text S4. Inventory of included study types

Included studies comprised original experimental work in animal models (rodent, gnotobiotic, humanized microbiota), in vitro systems (cell lines, organoids, colonic fermentation, ex vivo faecal incubations), and human subjects (controlled trials, cohort or cross-sectional studies); high-quality narrative, systematic, or integrative reviews; and regulatory or methodological publications on microbiome endpoints in food safety (EFSA, FDA, WHO).

Excluded were studies without microbiome-related endpoints or with endpoints not attributable to additives; studies focused only on macronutrient or caloric profiles; conference abstracts, editorials, and letters without primary data; studies using supra-physiological doses without any discussion of human relevance; and non-English publications. From each study, additive type and class, exposure dose and its relation to estimated human intake, study design and biological tier, microbiota endpoints, mechanistic pathways, host outcomes, and host-susceptibility variables were extracted. Given the heterogeneity of designs, a qualitative integrative synthesis organized by biological pathway was adopted, with mixture exposure and host susceptibility treated as cross-cutting themes.
